# Core Outcome Set-STAndardised Protocol Items: the COS-STAP Statement

**DOI:** 10.1186/s13063-019-3230-x

**Published:** 2019-02-11

**Authors:** Jamie J. Kirkham, Sarah Gorst, Douglas G. Altman, Jane M. Blazeby, Mike Clarke, Sean Tunis, Paula R. Williamson, for the COS-STAP Group, Adrian Aldcroft, Heather Bagley, Bronwen Connolly, Sarah Gorst, Jamie J. Kirkham, Tianjing Li, Karen Matvienko-Sikar, Victoria Thomas, Sean Tunis, Paula R. Williamson

**Affiliations:** 1 MRC North West Hub for Trials Methodology Research, Department of Biostatistics, University of Liverpool, Block F Waterhouse Building, 1-5 Brownlow Street, Liverpool, L69 3GL UK; 2 Centre for Statistics in Medicine, Nuffield Department of Orthopaedics, Rheumatology and Musculoskeletal Sciences, University of Oxford, Oxford, UK; 3 MRC ConDuCT II Hub for Trials Methodology Research, Population Health Sciences, University of Bristol, Bristol, UK; 4 Northern Ireland Hub for Trials Methodology Research, Centre for Public Health, Queen’s University Belfast, Belfast, UK; 5 Center for Medical Technology Policy, Baltimore, USA

**Keywords:** Core outcome set, Guideline, Protocol

## Abstract

**Background:**

Several hundred core outcome set (COS) projects have been systematically identified to date which, if adopted, ensure that researchers measure and report those outcomes that are most likely to be relevant to users of their research. The uptake of a COS by COS users will depend in part on the transparency and robustness of the methods used in the COS development study, which would be increased by the use of a standardised protocol. This article describes the development of the COS-STAP (Core Outcome Set-STAndardised Protocol Items) Statement for the content of a COS development study protocol.

**Methods:**

The COS-STAP Statement was developed following the Enhancing the Quality and Transparency Of Health Research (EQUATOR) Network’s methodological framework for guideline development. This included an initial item generation stage, a two-round Delphi survey involving more than 150 participants representing three stakeholder groups (COS developers, journal editors and patient and public involvement researchers interested in COS development), followed by a consensus meeting with eight voting participants.

**Results:**

The COS-STAP Statement consists of a checklist of 13 items considered essential documentation in a protocol, outlining the scope of the COS, stakeholder involvement, COS development plans and consensus processes.

**Conclusions:**

Journal editors and peer reviewers can use the guidance to assess the completeness of a COS development study protocol submitted for publication. By providing guidance for key content, the COS-STAP Statement will enhance the drafting of high-quality protocols and determine how the COS development study will be carried out.

**Electronic supplementary material:**

The online version of this article (10.1186/s13063-019-3230-x) contains supplementary material, which is available to authorized users.

## Introduction

Core outcome sets (COS), defined as an agreed standardised set of outcomes that should be measured and reported as a minimum [1], are being registered to support outcome choices in clinical trials, routine care [2] and systematic reviews [3]. The number of COS studies being published is increasing, but uptake of these cos in research has been variable. However, the importance of COS uptake has been acknowledged amongst key stakeholders including trialists [4], trial funders [5], clinical guideline developers [6], regulative authorities [7], payers [8] and industry [9]. Development of the COS-STAR (Core Outcome Set-STAndards for Reporting) guideline aims to improve the quality of reporting COS development studies [10].

The COS-STAD (Core Outcome Set-STAndards for Development) minimum standard recommendations have been previously identified as key considerations to improve the methodological approach towards planning a COS study [11]. Whilst making the protocol publicly available for a COS development study was not agreed upon as a COS-STAD minimum standard, it was suggested that the availability of a protocol would ensure that the methods are explicitly documented before the COS development project starts, thus promoting research integrity and transparency of the finalised COS. The availability of a protocol would also assist with the appraisal of a COS against the COS-STAD minimum standards and reduce arbitrary or informed decision-making during the consensus process. Changes in any of the methods-based minimum standards during the consensus process may lead to inappropriate decisions influencing the consensus process, particularly if these changes are made with some knowledge of the results. As an example, a change in the consensus definition following the analysis of outcome preference scores may lead to a different COS. This may affect the credibility of the COS and may deter COS users (e.g. trialists) from prioritising its adoption.

Few published COS listed in the COMET (Core Outcome Measures in Effectiveness Trials, www.comet-initiative.org) database have an associated publicly accessible protocol. In an ongoing review, only two out of 42 cancer COS studies identified in the COMET database published between 1981 and 2016 had a protocol, although neither of these protocols were published [12, 13]. However, a crude search of PubMed has identified a stark increase in the number of published COS protocols in more recent years, with 31 identified in 2017. The rarity of COS protocols may be due in part to the lack of knowledge about what to include in a protocol, given there is no current guidance. In response to this gap, we developed the COS-STAP (Core Outcome Set-STAndardised Protocol Items) Statement as a framework for COS developers to document their COS development plans. The checklist is relevant to any consensus method chosen (e.g. Delphi survey, expert panel meeting) or any combination of methods, but it considers only the part of the process that determines ‘what’ outcomes should be measured within a particular research or practice setting. Guidance about ‘how’ to select outcome measurement instruments exists [14], although this guidance does not include considerations for the protocol.

This paper summarises the development of the guideline, presents the COS-STAP Statement and provides an Explanation and Elaboration (E + E) supplement detailing the rationale and supporting evidence of the importance of each item, with examples from published COS protocols.

### Development of the COS-STAP Statement

The COS-STAP Statement was developed in consultation with three key stakeholder groups with complementary roles: COS developers, journal editors and those with interest in patient and public involvement (PPI) and participation in COS research (i.e. a separate subgroup of COS developers). As detailed in subsequent sections, the Statement was developed by generating an initial list of protocol items, undertaking a two-round Delphi survey and holding a consensus meeting—an approach that follows the Enhancing the Quality and Transparency Of Health Research (EQUATOR) Network’s methodological framework for guideline development [15].

#### Development of the preliminary list of protocol items

The process began with a preliminary checklist of 35 items derived from the personal experiences of COS development amongst the project management group (listed study authors). The COS-STAR reporting guideline [10] was also consulted when developing the initial list, because many protocol items would mirror applicable items at the reporting stage. The list of items was grouped into domains and subdomains to reflect the stages of planning a COS study (Additional file 1).

#### Delphi survey

This preliminary list of items was included in an international two-round Delphi survey in order to ascertain their importance. The survey participants included the three key stakeholder groups, chosen to encompass those who have experience in COS development or processing the publication of COS manuscripts. Personalised invitations by email were sent to (1) 288 lead investigators of a published COS or those involved with an ongoing COS study in the COMET database, with a request to forward the invitation to any methodologists involved; (2) editors-in-chief of 162 journals where COS studies have been published; and (3) ten PPI researchers involved with COS. The aim was to recruit as many individuals from each stakeholder group as possible for the Delphi exercise.

Delphi participants rated the importance of each item on a scale from 1 (not important) to 9 (critically important). In round 1 of the Delphi study, participants could suggest new items to be included in the second round, as well as make suggestions to improve the clarity of the wording of existing items (Additional file 1). In round 2, each participant who participated in round 1 was shown the number of respondents and distribution of scores for each item, for all stakeholder groups separately, together with their own score from round 1. An additional 65 items were suggested in round 1, ten of which were scored in round 2 following the removal of duplicate suggestions and review by the project management group (Additional file 2).

The consensus criteria were consistent with our other related guidance work [10, 11] and were defined a priori in the research plan submitted to the Ethics Committee. Consensus was achieved if at least 70% of the voting participants from each stakeholder group scored between 7 and 9. COS developers (*n* = 133), journal editors (*n* = 15) and COS PPI researchers (*n* = 10) participated in both rounds (Additional file 3); 121 were from Europe, 27 from the USA/Canada, six Oceania and four Asia. The variable number of respondents per stakeholder group did not affect the results since feedback in round 2 was presented by group. The Delphi process was conducted and managed using DelphiManager software developed by the COMET Initiative [16].

The consensus meeting was a half-day event held in London, UK, in July 2018 with eight international voting participants, including COS developers (*n* = 4), journal editors (*n* = 2) and COS PPI researchers (*n* = 2). Individuals were broadly selected based on completion of both rounds of the Delphi survey, COS development experience (completed versus ongoing developers) and, in the case of journal editors, those who regularly publish COS protocols. If an individual could not attend, he/she was replaced by someone else from the same stakeholder group wherever possible. Three UK participants attended the consensus meeting in person whilst the others (2 UK, 1 European and 2 USA participants) joined the meeting via Adobe Connect web conferencing software [17]. Two additional participants (one facilitator who chaired the meeting and one note taker) attended the meeting but did not participate in the discussion or voting.

The Delphi results for all 45 items were presented to the consensus meeting participants prior to the meeting (Additional file 3). At the start of the meeting, it was agreed by the participants that 13 items should be automatically included in the final COS-STAP checklist because each of these items reached consensus amongst all stakeholder groups in the Delphi survey, and all were included in the COS-STAR reporting guideline (Additional file 4). Seven items were excluded with limited discussion because less than 50% of participants from each stakeholder group thought the item was critical for inclusion, and none of these were included in the COS-STAR reporting guideline (Additional file 4).

The Delphi survey results from the remaining 25 items were then presented to the consensus meeting participants; each item was discussed and then voted on, and retained if more than 70% of the voting participants (i.e. at least 6 of the 8 voting participants) scored between 7 and 9. Voting was undertaken using Poll Everywhere [18], which allowed remote voting via a weblink. Results from the voting and key discussion points are presented in Additional file 4.

After all items were discussed, the meeting facilitator introduced one additional item (item 46) relating to how COS developers planned to disseminate results to study participants and COS users. This item was suggested by a number of participants in the first round of the Delphi survey, but due to an oversight it was not included in round 2. The consensus panel agreed to discuss and vote on this item, and consensus was achieved that it should be included in the Statement (Additional file 4).

During the consensus meeting, participants discussed the importance of distinguishing between items to be included in a published protocol, for which this guideline has been developed, and the additional items that are not pertinent for inclusion in the Statement but may still need to be considered in relation to the COS development process itself. Examples of the latter are described in the points below. The need to merge some items and create subitems was noted, together with suggestions for the explanatory document to enhance the usefulness of the final Statement. Specifically, the following amendments were discussed and agreed by the consensus meeting panel during the meeting:

1. Stakeholders. There were eight items under this topic, and whilst it was agreed that all could provide useful information, some were considered too specific to be included and four were voted out (item numbers 14, 16, 18, 20, Additional file 4). Of the four remaining items (item numbers 13, 15, 17, 19, Additional file 4), one item covering which stakeholder groups are to be involved in the COS development process (including rationale), and a description of how individuals from within each stakeholder group will be identified should be included. The E + E document will discuss the number of planned participants within each stakeholder group and any roles, inclusive of consensus participation or study design roles.
2. Outcome scoring/feedback summary. To include a summary of how participants would receive any feedback during the consensus process as part of how the consensus results.
3. Missing data. To include one item reflecting missing data in the Statement, with specific sources of missing data (attrition and partial responses) highlighted in the E + E document.
4. Ethics and informed consent. To combine aspects of research ethics committee (REC)/institutional review board (IRB) approvals and informed consent as a single item.

Following the meeting, a draft of the Statement was circulated to the project management group (study authors) and the remainder of the COS-STAP Group who participated in the consensus meeting. All comments and revisions were taken into consideration and the checklist revised accordingly. The process of obtaining feedback and refining the checklist was repeated until no further changes were needed.

### The COS-STAP Statement

The 13-item COS-STAP Statement presented in Table 1 applies to COS development studies where the aim of the study is to decide what outcomes should be included in the COS; it does not extend to cover plans on how those outcomes should be defined or measured. The items are categorised into six main sections: Title/Abstract, Introduction, Methods, Analysis, Ethics and Dissemination, Administrative Information.

**Table 1:** Core Outcome Set-STAndardised Protocol Items: the COS-STAP Statement

TITLE/ABSTRACT		
Title	1a	Identify in the title that the paper describes the protocol for the planned development of a COS
Abstract	1b	Provide a structured abstract
INTRODUCTION		
Background and objectives	2a	Describe the background and explain the rationale for developing the COS, and identify the reasons why a COS is needed and the potential barriers to its implementation
	2b	Describe the specific objectives with reference to developing a COS
Scope	3a	Describe the health condition(s) and population(s) that will be covered by the COS
	3b	Describe the intervention(s) that will be covered by the COS
	3c	Describe the context of use for which the COS is to be applied
METHODS		
Stakeholders	4	Describe the stakeholder groups to be involved in the COS development process, the nature of and rationale for their involvement and also how the individuals will be identified; this should cover involvement both as members of the research team and as participants in the study
Information sources	5a	Describe the information sources that will be used to identify the list of outcomes. Outline the methods or reference other protocols/papers
	5b	Describe how outcomes may be dropped/combined, with reasons
Consensus process	6	Describe the plans for how the consensus process will be undertaken
Consensus definition	7a	Describe the consensus definition
	7b	Describe the procedure for determining how outcomes will be added/combined/dropped from consideration during the consensus process
ANALYSIS		
Outcome scoring/feedback	8	Describe how outcomes will be scored and summarised, describe how participants will receive feedback during the consensus process
Missing data	9	Describe how missing data will be handled during the consensus process
ETHICS and DISSEMINATION		
Ethics approval/informed consent	10	Describe any plans for obtaining research ethics committee/institutional review board approval in relation to the consensus process and describe how informed consent will be obtained (if relevant)
Dissemination	11	Describe any plans to communicate the results to study participants and COS users, inclusive of methods and timing of dissemination
ADMINISTRATIVE INFORMATION		
Funders	12	Describe sources of funding, role of funders
Conflicts of interest	13	Describe any potential conflicts of interest within the study team and how they will be managed

The COS-STAP development process ensured that the items complement the COS-STAR reporting guideline whenever possible, facilitating the transition of a COS protocol to the COS final report. The COS-STAP Statement covers the minimum protocol items needed to be included in a published protocol and is designed to be applicable regardless of the planned consensus methodology (inclusive of mixed methods). The included items should capture all sources of potential deviations that may lead to inappropriate decisions influencing the consensus process. In certain circumstances, and at the discretion of the COS developers, additional details may be warranted for inclusion in the protocol in order to carry out the study as planned.

In the accompanying E + E document (Additional file 5), explanations are provided for the meaning and rationale for each protocol item, with examples provided from published COS protocols.

## Discussion

Despite the fact that a study protocol did not reach consensus as part of the COS-STAD minimum standards for COS development, it is still considered good research practice in general to develop a protocol before the start of a study and make it publicly available on a suitable platform. The COS-STAP Statement provides international consensus-based guidance on what information should be included in a published protocol for COS development studies. The checklist comprises 13 minimum items to be included in COS protocols to promote transparency. We also prepared the COS-STAP E + E document to provide an explanation of each of the COS-STAP Statement items and to provide good examples of how these items have been previously addressed in published COS protocols. We strongly recommend that COS developers consult the E + E guidance when developing their COS protocol using COS-STAP to optimise the study design.

During the development of the COS-STAP Statement, we ensured that there was adequate harmonization between key methodological items that were included in the COS-STAR reporting guideline. This ensures a smooth transition from a COS protocol into the final report of a COS study. Eleven of the 13 COS-STAP items have a corresponding COS-STAR item. Where it was felt that the wording, context or understanding of items could be improved from the COS-STAR Statement, we modified the text; e.g. the setting (a term used in COS-STAR) in which the COS is to be applied was commonly misunderstood by the consensus meeting panel, and it was felt that ‘context of use’ would be more appropriate. We also extended item 8 to include ‘how participants will receive feedback during the consensus process’. Whilst this may not be important for final COS reporting, it has been previously shown that different feedback approaches may influence the final core set [19], and therefore the planned feedback approach to be taken should be described in the COS protocol. More information on different methods of providing feedback and on COS development consensus methodology in general can be found in the *COMET Handbook* [20].

We added two new items to the COS-STAP Statement that were not included in COS-STAR. Item 9 related to how missing data would be handled during the consensus process. Similarly to the point made about item 8, missing data may lead to bias (and the final COS) if missing data is informed by the results, for example, if a participant decided not to respond in a round of Delphi because their opinions differed from their peers. Describing approaches for dealing with missing data (e.g. how to deal with views from participants who have not scored all outcomes) at the protocol stage will help avoid any arbitrary decisions that developers might be forced to make during the consensus process. Item 11 related to dissemination strategies for the study results, and it was introduced at the consensus meeting stage. The item was supported by the majority of consensus meeting participants and was a mandatory section for *BMJ Open* [21], a journal which publishes a large number of COS protocols.

COS-STAP focusses on the main methodological plans for developing a COS. However, during the discussion of dissemination plans, the importance of COS implementation was raised as being relevant to the development process, which might include both the intended audience and users of COS (e.g. trialists) as stakeholders, and to the pathways to reach these stakeholders. The consensus meeting group decided that developing an implementation plan was a separate issue, but at the COS development stage, COS developers should consider how the development might underpin the subsequent implementation of the COS. For this reason it was agreed that any potential barriers to COS implementation should be discussed (item 2a). For example, the location of many participants involved in COS development has primarily been in North America and Europe [22], and this may affect the perceived relevance of the COS in groups who are underrepresented, such as those from Latin America, Asia Pacific, Africa and low- and middle-income countries, and therefore potentially subsequent uptake.

The study had two limitations. The participation rate in the international Delphi survey appeared to be low, particularly for journal editors, although retention in round 2 was above 85% overall, with no evidence of attrition bias. Due to logistical constraints in arranging a consensus meeting, only a small group of eight participants voted on the items. To address this limitation, we structured the meeting such that items that reached the ‘consensus in’ criteria for the larger group of Delphi participants for all stakeholder groups were dealt with collectively, and it was decided that all of these should be included in the final COS-STAP Statement. Those unable to attend the meeting also had the opportunity to comment on the final items and processes of the meeting.

The success of the COS-STAP Statement requires the support of COS developers utilising the guideline. COS developers who register their study on the COMET database will be alerted to the existence of the COS-STAP Statement. We anticipate that widespread adoption of the COS-STAP Statement will facilitate improvement in the content of COS development studies and provide editorial support for assessing the completeness of submitted COS protocols for publication. Readers are invited to submit comments, criticisms, experiences and recommendations via the COMET website (http://www.comet-initiative.org/contactus), which will be considered for future refinement of the COS-STAP Statement.

## Additional files

Additional file 1: Preliminary checklist of 35 items derived from the personal experiences of COS development by the project management group. (DOCX 34 kb)

Additional file 2: Additional items suggested by Delphi participants in round 1. (DOCX 31 kb)

Additional file 3: Consensus matrix for round 1 and round 2 of the COS-STAP Delphi survey. (DOCX 59 kb)

Additional file 4: COS-STAP consensus meeting results/discussion. (DOCX 42 kb)

Additional file 5: Explanation and Elaboration document for COS-STAP. (DOCX 253 kb)

## Abbreviations

COMET: Core Outcome Measures in Effectiveness Trials
COS: Core outcome set
COS-STAD: Core Outcome Set-STAndards for Development
COS-STAP: Core Outcome Set-STAndardised Protocol Items
COS-STAR: Core Outcome Set-STAndards for Reporting
EQUATOR: Enhancing the QUAlity and Transparency Of health Research
PPI: Patient and public involvement

## References

[1] Williamson PR, Altman DG, Blazeby JM, Clarke M, Devane D, Gargon E (2012). Developing core outcome sets for clinical trials: issues to consider. Trials.

[2] Davis K, Gorst SL, Harman N, Smith V, Gargon E, Altman DG (2018). Choosing important health outcomes for comparative effectiveness research: an updated systematic review and involvement of low and middle income countries. PLOS One.

[3] Clarke M, Williamson PR (2016). Core outcome sets and systematic reviews. Systematic Reviews.

[4] Chan A-W, Tetzlaff JM, Altman DG, Laupacis A, Gøtzsche PC, Krleža-Jerić K (2013). SPIRIT 2013 Statement: defining standard protocol items for clinical trials. Ann Intern Med.

[5] National Institute for Health Research Guidance Notes 2017. [ONLINE]. Available at: https://www.nihr.ac.uk/funding-and-support/documents/current-funding-opportunities/hta/hta-stage-1-guidance-notes.pdf. Accessed 28 June 2018.

[6] National Institute for Health and Care Excellence: Developing NICE guidelines: the manual 2017. [ONLINE]. Available at: https://www.nice.org.uk/process/pmg20/chapter/developing-review-questions-and-planning-the-evidence-review. Accessed 28 June 2018. 26677490

[7] European Medicines Agency: Guideline on the clinical investigation of medicinal products for the treatment of asthma 2015. [ONLINE]. Available at: http://www.ema.europa.eu/docs/en_GB/document_library/Scientific_guideline/2015/12/WC500198877.pdf. Accessed 28 June 2018.

[8] Tunis SR, Maxwell LJ, Graham ID, Shea BJ, Beaton DE, Bingham CO (2017). Engaging stakeholders and promoting uptake of OMERACT core outcome instrument sets. J Rheumatol.

[9] European Federation of Pharmaceutical Industries and Associations: Healthier future - the case for outcomes-based, sustainable healthcare 2016. [ONLINE]. Available at: https://www.efpia.eu/about-medicines/use-of-medicines/outcomes-focused-sustainable-healthcare/. Accessed 28 June 2018.

[10] Kirkham JJ, Gorst S, Altman DG, Blazeby JM, Clarke M, Devane D (2016). Core Outcome Set – Standards for Reporting: the COS-STAR Statement. PLOS Med.

[11] Kirkham JJ, Davis K, Altman DG, Blazeby JM, Clarke M, Tunis S, Williamson PR (2017). Core Outcome Set STAnDards for Development: the COS-STAD Recommendations. PLOS Med.

[12] McNair AGK, Whistance RN, Forsythe RO, Macefield R, Rees J, Pullyblank AM (2016). Core Outcomes for Colorectal Cancer Surgery: a Consensus Study. PLOS Med.

[13] Gerritsen A, Jacobs M, Henselmans I, Van Hattum J, Efficace F, Creemers G-J (2016). Developing a core set of patient-reported outcomes in pancreatic cancer: a Delphi survey. Eur J Cancer.

[14] Prinsen CAC, Vohra S, Rose MR, Boers M, Tugwell P, Clarke M (2016). How to select outcome measurement instruments for outcomes included in a “Core Outcome Set” – a practical guideline. Trials.

[15] Moher D, Schulz KF, Simera I, Altman DG (2010). Guidance for developers of health research reporting guidelines. PLOS Med.

[16] COMET Initiative Delphi Manager 2017. [ONLINE]. Available at: http://www.comet-initiative.org/delphimanager/. Accessed 15 May 2017.

[17] Adobe Connect [ONLINE]. Available at https://www.adobe.com/products/adobeconnect.html. Accessed 28 June 2018.

[18] Poll Everywhere [ONLINE]. https://www.polleverywhere.com/. Accessed 28 June 2018.

[19] Brookes ST, Macefield RC, Williamson PR, McNair AG, Potter S, Natalie S (2016). Three nested randomized controlled trials of peer-only or multiple stakeholder group feedback within Delphi surveys during core outcome and information set development. Trials.

[20] Williamson PR, Altman DG, Bagley H, Barnes KL, Blazeby JM, Brookes ST (2017). The COMET Handbook: version 1.0. Trials.

[21] BMJ OPEN Authors [ONLINE]. https://bmjopen.bmj.com/pages/authors/#study_protocols. Accessed 28 June 2018.

[22] Williamson PR. 26^th^ Bradford Hill Memorial Lecture. Improving health by improving trials: from outcomes to recruitment and back again [ONLINE] Available at: https://www.lshtm.ac.uk/newsevents/events/26th-bradford-hill-memorial-lecture. Accessed 30 August 2017.

