# Supplementary material for: Core Outcome Set-STAndardised Protocol Items: the COS-STAP Statement
Source: Trials. 2019 Feb 11;20:116. doi: 10.1186/s13063-019-3230-x (PMC6371434; doi:10.1186/s13063-019-3230-x)
Supplement: Supplementary file 1 — Preliminary checklist of 35 items derived from the personal experiences of COS development by the project management group. (DOCX 34 kb) [file 13063_2019_3230_MOESM1_ESM.docx]

Additional file 1: Preliminary checklist of 35 items derived from the personal experiences of COS development by the project management group. [Text in red indicates modifications to the wording of items following Delphi participant comments in Round 1 – the modified text was then used in Round 2]

| **Item Number** | **Name** | **Domain [sub-domain]** |
| --- | --- | --- |
| 1 | Identify in the title that the paper describes the protocol for the planned development of a COS | TITLE/ABSTRACT  [Title] |
| 2 | Provide a structured abstract | TITLE/ABSTRACT  [Abstract] |
| 3 | Describe the background and explain the rationale for developing the COS  [*Describe the background and explain the rationale for developing the COS, and identify the reasons why a COS is needed and the potential barriers to its implementation*] | INTRODUCTION  [Background and Rationale] |
| 4 | Describe the specific objectives with reference to developing a COS | INTRODUCTION  [Objectives] |
| 5 | Describe the health condition(s) and population(s) that will be covered by the COS | INTRODUCTION  [Scope – Health Condition and Population] |
| 6 | Describe the intervention(s) that will be covered by the COS | INTRODUCTION  [Scope – Intervention] |
| 7 | Describe the setting(s) that will be covered by the COS | INTRODUCTION  [Scope - Setting] |
| 8 | Indicate the COS study registration details and registry name. If not yet registered indicate the intended registry | ADMINISTRATIVE INFORMATION  [Study Registration] |
| 9 | Describe any study oversight committees  [*Describe any study oversight committees including their stakeholder groups and role*] | ADMINISTRATIVE INFORMATION  [Oversight committees] |
| 10 | Describe sources of funding, role of funders | ADMINISTRATIVE INFORMATION  [Funding] |
| 11 | Describe any potential conflicts of interest within the study team and how these will be managed | ADMINISTRATIVE INFORMATION  [Conflicts of Interest] |
| 12 | Describe the stakeholder groups to be involved in the COS development process and the rationale for their involvement | METHODS –STAKEHOLDERS  [Participants - Groups] |
| 13 | Describe the eligibility criteria for individuals from each stakeholder group | METHODS –STAKEHOLDERS  [Participants - Eligibility] |
| 14 | Describe how individuals of each stakeholder groups will be identified | METHODS –STAKEHOLDERS  [Participants - Identification] |
| 15 | Describe how individuals of each stakeholder group will be chosen from within the stakeholder group  [*Describe whether all eligible individuals within a stakeholder group will be invited to take part or whether some form of selection will be used*] | METHODS –STAKEHOLDERS  [Participants - Selection] |
| 16 | Describe how many planned individuals within each stakeholder group will be invited to participate in the consensus process | METHODS –STAKEHOLDERS  [Participants – Sample Size] |
| 17 | Describe how individuals will be invited to take part in the consensus process | METHODS –STAKEHOLDERS  [Participants – Invitation] |
| 18 | Describe the information sources that will be used to identify the list of outcomes. Outline the methods or reference other protocols/papers. | METHODS –INFORMATION SOURCES [Information Sources] |
| 19 | Describe how outcomes may be dropped/combined, with reasons | METHODS –INFORMATION SOURCES  [Dropping/Combining] |
| 20 | Describe the methods to identify outcome descriptor terms | METHODS –INFORMATION SOURCES  [Descriptors] |
| 21 | Describe the plans for how the consensus process will be undertaken | METHODS [Consensus Process] |
| 22 | Describe what information will be presented to participants at the start of the consensus process | METHODS [Consensus Process] |
| 23 | Describe what each participant will be asked to do at each stage of the consensus process | METHODS [Consensus Process] |
| 24 | Describe how the participants will receive any feedback during the consensus process | METHODS [Consensus Process] |
| 25 | Describe how non-response (or partial response) will be handled during the consensus process | METHODS [Consensus Process] |
| 26 | Describe how the study material will be made patient friendly and understandable (if relevant)  [*Describe how the study material will be tailored for stakeholder groups such that it is understandable*] | METHODS [Consensus Process] |
| 27 | Describe the consensus definition | METHODS [Consensus Definition] |
| 28 | Describe the procedure for determining how outcomes will be added/combined/dropped from consideration during the consensus process | METHODS [Consensus Definition] |
| 29 | Describe how outcomes will be scored and summarised | ANALYSIS [Outcome Scoring] |
| 30 | Describe how the response rate will be maximised | ANALYSIS [Response Rate] |
| 31 | Describe how attrition bias will be assessed | ANALYSIS [Attrition] |
| 32 | Describe any software that will be used during the consensus process and to analyse the results | ANALYSIS [Software] |
| 33 | Describe any plans for obtaining research ethics committee / institutional review board approval in relation to the consensus process (if relevant) | ETHICS/ DISSEMINATION [Ethics Approval] |
| 34 | Describe how informed consent will be obtained (if relevant) | ETHICS/ DISSEMINATION [Informed Consent] |
| 35 | Describe any details about how the confidentiality of data collection will be preserved during the consensus process (if relevant) | ETHICS/ DISSEMINATION [Confidentiality of Data] |
