# Supplementary material for: Core Outcome Set-STAndardised Protocol Items: the COS-STAP Statement
Source: Trials. 2019 Feb 11;20:116. doi: 10.1186/s13063-019-3230-x (PMC6371434; doi:10.1186/s13063-019-3230-x)
Supplement: Supplementary file 2 — Additional items suggested by Delphi participants in round 1. (DOCX 31 kb) [file 13063_2019_3230_MOESM2_ESM.docx]

Additional file 2: Additional items suggested by Delphi participants in Round 1. [Text in red indicates items included in Round 2 of the Delphi following discussion with the project management group]

| **Additional item suggestions** | **Item Number** | **Project management group decision** |
| --- | --- | --- |
|  |  |  |
| Describe how patients have been involved in helping design the COS study | 1 | NEW ITEM: Describe how various stakeholders have been involved in helping design the COS study |
| Describe how patients will be involved in the oversight of the study | 2 | Covered by existing item 9 (oversight committees) |
| Describe how participants will get to hear of the study results | 3 | *This item was revisited at the consensus meeting.* |
| Through which processes will outcomes be identified (lit review; patient perspective interviews...) | 4 | Covered by existing item 18 (information sources that will be used) |
| COS dissemination | 5 | Same as additional item 3 |
| how will the final COS be agreed upon (face-to-face meeting...) | 6 | Covered by existing item 21 (description of consensus process) |
| Details of any consensus meeting; who will attend how they will be invited/selected; how many; from which stakeholder groups; equal weighting | 7 | Covered by existing item 21 (description of consensus process), make clear covers all aspects of consensus procedure |
| Define perspectives to be studied - patient; biologic; researcher; society | 8 | Covered by existing item 12 (stakeholders involved) |
| describe the rationale and foundation for each proposed core outcome | 9 | Does not make sense to include at protocol stage - beyond protocol |
| Describe the proposed number of Delphi participants | 10 | Covered by existing item 16 (how many planned individuals within each stakeholder group) |
| Describe the proposed dissemination plan for the COS | 11 | Same as additional item 3 |
| Plans for validating the COS have been described (is this COS useful?) | 12 | NEW ITEM: Describe any plans for validating the COS |
| Plans for disseminating the COS have been described (do people know to use this COS?) | 13 | Same as additional item 3 |
| Barriers to implementation of the COS have been considered prospectively. | 14 | Covered by existing item 3 (Background). |
| Describe the maintenance plan and measures | 15 | Does not make sense to include at protocol stage - beyond protocol |
| describe instructions or methods for maintaining anonymity if applicable to the consensus method being used (eg Delphi) | 16 | Covered by existing item 35 (confidentiality of data collection) |
| provide instructions for how the perspective that members should be taking in voting (eg their personal perspective or perspective of any organization that they are representing) | 17 | Covered by existing item 22 (information presented to participants) |
| definition of concepts being discussed/voted upon (e.g.; definition of a "domain") | 18 | Covered by existing item 22 (information presented to participants) |
| description of the desired proportion of consensus panel from each key stakeholder group and justification for this | 19 | NEW ITEM: Describe the rationale for the desired proportion from each stakeholder group for each component of the consensus process Proportion covered by existing item 16 but rationale is not. |
| description of the goal sample size for the consensus panel and justification for this | 20 | Same as additional item 19 |
| description of how potential conflict of interest among invited participants will be identified and dealt with | 21 | NEW ITEM: Describe how potential conflicts of interest among invited participants will be identified and dealt with |
| description of how items being voted upon/discussed will be ordered for presentation to participants (eg; randomized order; alphabetical order) | 22 | NEW ITEM: Describe how items being voted upon/discussed will be ordered for presentation to participants (e.g.  randomized order; alphabetical order; grouped according to an outcome classification framework or domains; frequency of use in previous clinical trials) |
| description of how any qualitative responses from participants will be shared in subsequent rounds of the consensus process | 23 | Covered by existing item 24 (description of feedback received). |
| description of period of time allowed for response to each round of the consensus process | 24 | Covered by existing item 21 (description of consensus process) |
| Plans for dealing with high scores from most participants for all items in a traditional Delphi so that no items can be dropped in the 2nd round | 25 | Covered by existing item 28 (procedures for dropping outcome) |
| How protocol amendments will be agreed | 26 | Related to existing item 9 |
| Whether there is wiggle room in the a priori criterion for consensus (e.g. if 70%; is 69.5% consensus or not?) | 27 | Would normally round up but related existing item 9 |
| Whether consensus will be determined separately for different stakeholder groups (and if so why) | 28 | Covered by existing item 27 (consensus definition) |
| report any outcome classification framework used at SR level | 29 | Related to additional item 22 |
| how steering committee debriefs during process will be conducted and what will be presented | 30 | Covered by existing item 9 (oversight committees) |
| A section detailing any modifications to the outcome dropping and why this was needed | 31 | Relates to reporting rather than protocol |
| A statement of support by the stakeholders for the COS. ie. at the end of the COS x% of participants agreed to support this COS | 32 | NEW ITEM: Describe any plans for obtaining a statement of support by the stakeholders for the COS (e.g. at the end of the COS x% of participants agreed to support this COS) |
| Self-assessment of risk of bias at the various stages of COS development | 33 | Main aspects of bias already covered (Attrition – existing Item 31) |
| Details on elicitation of preferences from stakeholders (independent of the systematic review) through Focus group interviews | 34 | Covered by existing item 18 (information sources that will be used) |
| Describe the number of outcomes that the investigators expect to include in the final COS | 35 | NEW ITEM: Describe whether the investigators expect to include a particular number of outcomes in the final COS |
| Dissemination plans for COS | 36 | Same as additional item 3 |
| Describe which characteristics of the participants of the consensus procedure (including patients!) will be collected | 37 | NEW ITEM: Describe which participant characteristics will be collected |
| what is the criteria for deciding which outcome domains will be passed for discussion to the face-to-face consensus meeting | 38 | Covered by existing item 21 (description of consensus process) |
| desc ribe plans for review of the COS | 39 | NEW ITEM: Describe plans for subsequent review of the COS |
| Explain if there will be consensus meetings or only internet-based survey; ... | 40 | Covered by existing item 21 (description of consensus process) |
| Planned publication / dissemination / international recognition of the COS | 41 | Same as additional item 3 |
| Details of preliminary literature search on existing COS / instruments for the target patients | 42 | Covered by existing item 3 (Background) |
| Details of the executive committee members making decisions | 43 | Covered by existing item 9 (Oversight committees) |
| Proposed plans to disseminate the COS | 44 | Same as additional item 3 |
| The plans for feeding back the results of the study to all participants including patients once the COS has been agreed. | 45 | Same as additional item 3 |
| Neuropsichiatric Inventory Dementia Patient | 46 | Exclude |
| Any unique aspects of the intervention that may require "new" or "non-traditional/non-legacy" outcomes | 47 | Covered by existing item 3 (Background) |
| State whether or not there is already a core set for the disease; if so how will this be different (different intervention; different population; etc) | 48 | Covered by existing item 3 (Background) |
| Describe countries/parts of the world involved with justification (for generalizability) | 49 | Covered by existing item 12 (stakeholders involved and rationale for including them) |
| describe what you will do with the outcomes that reached consensus in 1st round; proceeding in the 2nr round | 50 | Covered by existing item 21 (description of consensus process) |
| describe your consensus definition taking to account possible differences in consensus between stakeholder groups | 51 | Covered by existing item 27 (consensus definition) |
| describe other methods you are planning to use for consensus (Delphi/Consensus meeting/steering group decisions) and the weight of these methods for reaching final consensus | 52 | Covered by existing item 21 (description of consensus process) |
| Provide information on data sharing (ICMJE requirement) | 53 | NEW ITEM: Provide information on data sharing |
| Engage patients in the research from the beginning as partners in the research | 54 | Same as additional items 2 and 3 |
| Engage patients as patient researchers to supplement standard researchers | 55 | Same as additional items 2 and 3 |
| Engage patients in the research report writing; and follow up communications | 56 | Same as additional items 2 and 3 |
| Clearly define the scope of the core outcome set | 57 | Covered by existing items 5-7 (Scope) |
| Explain how stakeholders have been selected to ensure cross-cultural or international COS relevance | 58 | Covered by existing item 12 (stakeholders involved and rationale for including them) |
| Describe how the results of the consensus process will be used in the final selection process | 59 | Covered by existing item 21 (description of consensus process) |
| Describe how the final outcomes will be selected for the core outcome set | 60 | Covered by existing item 21 (description of consensus process) |
| Describe how measurement instruments for each outcome will be selected | 61 | Not a protocol for the 'how' to measure outcomes |
| Dissemination plans | 62 | Same as additional item 3 |
| Describe the process by which the scope of the COS may be decided or adapted; if at all | 63 | Covered by existing item 9 (oversight committees and their roles, e.g. agreeing protocol amendments) |
| Describe plans for disseminating; and promoting use of; the core outcome set | 64 | Same as additional item 3 |
| Describe patient and public involvement in the design; planning and implementation of the COS (and as distinct from participation of the public/stakeholders) | 65 | Same as additional items 2 and 3 |
