# Supplementary material for: Core Outcome Set-STAndardised Protocol Items: the COS-STAP Statement
Source: Trials. 2019 Feb 11;20:116. doi: 10.1186/s13063-019-3230-x (PMC6371434; doi:10.1186/s13063-019-3230-x)
Supplement: Supplementary file 3 — Consensus matrix for round 1 and round 2 of the COS-STAP Delphi survey. (DOCX 59 kb) [file 13063_2019_3230_MOESM3_ESM.docx]

CONSENSUS IN (shaded in green)

Additional file 3: Consensus matrix for round 1 and round 2 of the COS-STAP Delphi survey

Consensus definition: Support from at least 70% of participants scoring ‘Critical’, i.e. score 7-9 [from a 1-9 scale]. Participants were excluded from the calculations (denominators) if they did not score on an item [overall retention in round 2 = 86%]

| Protocol Item | Round 1 | | | Round 2 | | |
| --- | --- | --- | --- | --- | --- | --- |
|  | COS developer (n=154) | Journal Editor (n=20) | Patient and public involvement representative (n=10) | COS developer (n=133)  [86% retention] | Journal Editor (n=15)  [65% retention] | Patient and public involvement representative (n=10) [100% retention] |
| Identify in the title that the paper describes the protocol for the planned development of a COS | | | | | | |
|  | 88% | 68% | 80% | 95% | 87% | 90% |
| Provide a structured abstract | | | | | | |
|  | 71% | 95% | 100% | 86% | 100% | 100% |
| Describe the background and explain the rationale for developing the COS | | | | | | |
| *Round 2 Wording: Describe the background and explain the rationale for developing the COS, and identify the reasons why a COS is needed and the potential barriers to its implementation* | | | | | | |
|  | 86% | 90% | 80% | 92% | 100% | 100% |
| Describe the specific objectives with reference to developing a COS | | | | | | |
|  | 83% | 100% | 80% | 89% | 100% | 100% |
| Describe the health condition(s) and population(s) that will be covered by the COS | | | | | | |
|  | 93% | 100% | 100% | 98% | 100% | 100% |
| Describe the intervention(s) that will be covered by the COS | | | | | | |
|  | 75% | 85% | 100% | 79% | 87% | 100% |
| Describe the setting(s) that will be covered by the COS | | | | | | |
|  | 68% | 75% | 80% | 68% | 80% | 80% |
| Indicate the COS study registration details and registry name. If not yet registered indicate the intended registry | | | | | | |
|  | 49% | 70% | 30% | 41% | 67% | 20% |
| Describe any study oversight committees | | | | | | |
| *Round 2 Wording: Describe any study oversight committees including their stakeholder groups and role* | | | | | | |
|  | 33% | 50% | 20% | 18% | 27% | 10% |
| Describe sources of funding; role of funders | | | | | | |
|  | 64% | 95% | 40% | 66% | 100% | 60% |
| Describe any potential conflicts of interest within the study team and how these will be managed | | | | | | |
|  | 74% | 90% | 40% | 79% | 93% | 60% |
| Describe the stakeholder groups to be involved in the COS development process and the rationale for their involvement | | | | | | |
|  | 93% | 90% | 70% | 95% | 100% | 90% |
| Describe the eligibility criteria for individuals from each stakeholder group | | | | | | |
|  | 62% | 75% | 40% | 57% | 73% | 40% |
| Describe how individuals of each stakeholder groups will be identified | | | | | | |
|  | 60% | 55% | 20% | 46% | 53% | 10% |

| Protocol Item | Round 1 | | | Round 2 | | |
| --- | --- | --- | --- | --- | --- | --- |
|  | COS developer (n=154) | Journal Editor (n=20) | Patient and public involvement representative (n=10) | COS developer (n=133)  [86% retention] | Journal Editor (n=15)  [65% retention] | Patient and public involvement representative (n=10) [100% retention] |
| Describe how individuals of each stakeholder group will be chosen from within the stakeholder group | | | | | | |
| *Round 2 Wording: Describe whether all eligible individuals within a stakeholder group will be invited to take part or whether some form of selection will be used* | | | | | | |
|  | 48% | 63% | 30% | 38% | 53% | 20% |
| Describe how many planned individuals within each stakeholder group will be invited to participate in the consensus process | | | | | | |
|  | 55% | 55% | 40% | 43% | 47% | 30% |
| Describe how individuals will be invited to take part in the consensus process | | | | | | |
|  | 53% | 50% | 30% | 37% | 40% | 30% |
| Describe the information sources that will be used to identify the list of outcomes. Outline the methods or reference other protocols/papers | | | | | | |
|  | 91% | 89% | 80% | 96% | 93% | 90% |
| Describe how outcomes may be dropped/combined; with reasons | | | | | | |
|  | 80% | 70% | 80% | 83% | 87% | 90% |
| Describe the methods to identify outcome descriptor terms | | | | | | |
|  | 53% | 70% | 67% | 50% | 73% | 70% |
| Describe the plans for how the consensus process will be undertaken | | | | | | |
|  | 92% | 75% | 90% | 97% | 93% | 100% |
| Describe what information will be presented to participants at the start of the consensus process | | | | | | |
|  | 62% | 65% | 80% | 65% | 60% | 80% |
| Describe what each participant will be asked to do at each stage of the consensus process | | | | | | |
|  | 62% | 55% | 90% | 62% | 60% | 90% |
| Describe how the participants will receive any feedback during the consensus process | | | | | | |
|  | 66% | 50% | 90% | 60% | 47% | 80% |
| Describe how non-response (or partial response) will be handled during the consensus process | | | | | | |
|  | 62% | 50% | 80% | 53% | 60% | 70% |
| Describe how the study material will be made patient friendly and understandable (if relevant) | | | | | | |
| *Round 2 Wording: Describe how the study material will be tailored for stakeholder groups such that it is understandable* | | | | | | |
|  | 50% | 50% | 90% | 48% | 40% | 90% |
| Describe the consensus definition | | | | | | |
|  | 94% | 85% | 80% | 98% | 93% | 90% |
| Describe the procedure for determining how outcomes will be added/combined/dropped from consideration during the consensus process | | | | | | |
|  | 88% | 75% | 78% | 95% | 100% | 89% |
| Describe how outcomes will be scored and summarised | | | | | | |
|  | 88% | 85% | 80% | 94% | 93% | 80% |
| Describe how the response rate will be maximised | | | | | | |
|  | 44% | 70% | 30% | 32% | 73% | 10% |
| Describe how attrition bias will be assessed | | | | | | |
|  | 52% | 70% | 40% | 41% | 73% | 10% |
| Protocol Item | Round 1 | | | Round 2 | | |
|  | COS developer (n=154) | Journal Editor (n=20) | Patient and public involvement representative (n=10) | COS developer (n=133)  [86% retention] | Journal Editor (n=15)  [65% retention] | Patient and public involvement representative (n=10) [100% retention] |
| Describe any software that will be used during the consensus process and to analyse the results | | | | | | |
|  | 38% | 60% | 20% | 26% | 60% | 10% |
| Describe any plans for obtaining research ethics committee / institutional review board approval in relation to the consensus process (if relevant) | | | | | | |
|  | 61% | 80% | 50% | 63% | 93% | 50% |
| Describe how informed consent will be obtained (if relevant) | | | | | | |
|  | 54% | 80% | 70% | 53% | 87% | 70% |
| Describe any details about how the confidentiality of data collection will be preserved during the consensus process (if relevant) | | | | | | |
|  | 47% | 90% | 80% | 49% | 100% | 60% |
| ADDITIONAL ITEMS SCORED IN ROUND 2 ONLY | | | | | | |
| Provide information on data sharing | | | | | | |
|  |  |  |  | 17% | 60% | 38% |
| Describe how the various stakeholders have been involved in helping design the study | | | | | | |
|  |  |  |  | 19% | 40% | 78% |
| Describe the rationale for the desired proportion from each stakeholder group for each component of the consensus process | | | | | | |
|  |  |  |  | 22% | 13% | 38% |
| Describe any plans for validating the COS | | | | | | |
|  |  |  |  | 30% | 67% | 33% |
| Describe how items being voted upon/discussed will be ordered for presentation to participants | | | | | | |
|  |  |  |  | 26% | 40% | 22% |
| Describe any plans for obtaining a statement of support by the stakeholders for the COS | | | | | | |
|  |  |  |  | 26% | 20% | 11% |
| Describe whether the investigators expect to include a particular number of outcomes in the final COS | | | | | | |
|  |  |  |  | 22% | 20% | 0% |
| Describe which participant characteristics will be collected | | | | | | |
|  |  |  |  | 27% | 53% | 33% |
| Describe plans for subsequent review of the COS | | | | | | |
|  |  |  |  | 27% | 40% | 22% |
| Describe how potential conflicts of interest among invited participants will be identified and dealt with | | | | | | |
|  |  |  |  | 42% | 87% | 44% |
