# Supplementary material for: Core Outcome Set-STAndardised Protocol Items: the COS-STAP Statement
Source: Trials. 2019 Feb 11;20:116. doi: 10.1186/s13063-019-3230-x (PMC6371434; doi:10.1186/s13063-019-3230-x)
Supplement: Supplementary file 4 — COS-STAP consensus meeting results/discussion. (DOCX 42 kb) [file 13063_2019_3230_MOESM4_ESM.docx]

Additional file 4: COS-STAP consensus meeting results/discussion: Number and percentages of participants scoring each item as critical for inclusion (7-9). Items were voted into the guideline if 70% or more of the voting participants voted 7-9. Any textual suggested changes to the items are highlighted in blue.

| **Item No.** |  |  |
| --- | --- | --- |
| **1** | **TITLE:** Identify in the title that the paper describes the protocol for the planned development of a COS | ^*^Automatically voted in |
| **2** | **ABSTRACT:** Provide a structured abstract | ^*^Automatically voted in |
| **3** | **INTRODUCTION/BACKGROUND:** Describe the background and explain the rationale for developing the COS, and identify the reasons why a COS is needed and the potential barriers to its implementation | ^*^Automatically voted in |
| **4** | **INTRODUCTION/OBJECTIVES:** Describe the specific objectives with reference to developing a COS | ^*^Automatically voted in |
| **5** | **INTRODUCTION/HEALTH CONDITION-POPULATION:** Describe the health condition(s) and population(s) that will be covered by the COS | ^*^Automatically voted in |
| **6** | **INTRODUCTION/INTERVENTIONS:** Describe the intervention(s) that will be covered by the COS | ^*^Automatically voted in |
| **7** | **INTRODUCTION/SETTING:** Describe the setting(s) [change setting to ‘context of use’] that will be covered by the COS (e.g. for application in research studies; for use in routine care) | (7/8) 88% IN |
| **8** | **ADMINISTRATIVE INFORMATION/REGISTRATION:** Indicate the COS study registration details and registry name. If not yet registered indicate the intended registry | (3/7) 43% OUT |
| **9** | **ADMINISTRATIVE INFORMATION/OVERSIGHT COMMITTEES:** Describe any study oversight committees including their stakeholder groups and role | ^**^Automatically voted out |
| **10** | **ADMINISTRATIVE INFORMATION/FUNDERS:** Describe sources of funding; role of funders | (8/8) 100% IN |
| **11** | **ADMINISTRATIVE INFORMATION/CONFLICTS OF INTEREST TEAM:** Describe any potential conflicts of interest within the study team and how these will be managed | (7/8) 88% IN |
| **12** | **ADMINISTRATIVE INFORMATION/DATA SHARING:** Provide information on data sharing | (0/7) 0% OUT |
| **13** | **METHODS/STAKEHOLDER GROUPS:** Describe the stakeholder groups to be involved in the COS development process and the rationale for their involvement [the explanation and elaboration information should incorporate involvement in terms of the design of the COS study and participation in the consensus process as well as how many planned individuals within each stakeholder group] | ^*^Automatically voted in |
| **14** | **METHODS/STAKEHOLDER ELIGIBILITY:** Describe the eligibility criteria for individuals from each stakeholder group | (5/8) 63% OUT |
| **15** | **METHODS/STAKEHOLDER IDENTIFICATION:** Describe how individuals of each stakeholder groups will be identified [Should be considered in the same way as COS-STAR as part of ‘Stakeholder Group’ item] | Agreed to include as part of a previous item only |
| **16** | **METHODS/STAKEHOLDER SELECTION:** Describe whether all eligible individuals within a stakeholder group will be invited to take part or whether some form of selection will be used | (0/7) 0% OUT |
| **17** | **METHODS/STAKEHOLDER NUMBERS**: Describe how many planned individuals within each stakeholder group will be invited to participate in the consensus process [Combine with stakeholder group item and discuss in explanation and elaboration only] | Agreed to discuss in E+E as part of stakeholder item only |
| **18** | **METHODS/STAKEHOLDER INVITATION**: Describe how individuals will be invited to take part in the consensus process | ^**^Automatically voted out |
| **19** | **METHODS/STAKEHOLDER STUDY DESIGN**: Describe how the various stakeholders have been involved in helping design the study [see above – it was agreed that the design part should also be considered as part of the of the ‘Stakeholder Group’ item which also includes how stakeholders participated within the consensus process – to discuss in E+E only] | Agreed to discuss in E+E as part of stakeholder item only |
| **20** | **METHODS/STAKEHOLDER PROPORTION RATIONALE** : Describe the rationale for the desired proportion from each stakeholder group for each component of the consensus process | ^**^Automatically voted out |
| **21** | **METHODS/INFORMATION SOURCES:** Describe the information sources that will be used to identify the list of outcomes. Outline the methods or reference other protocols/papers | ^*^Automatically voted in |
| **22** | **METHODS/INFORMATION SOURCES/DROP-COMBINED:** Describe how outcomes may be dropped/combined; with reasons | ^*^Automatically voted in |
| **23** | **METHODS/INFORMATION SOURCES/DESCRIPTOR:** Describe the methods to identify outcome descriptor terms | (5/8) 63% OUT |
| **24** | **METHODS/CONSENSUS PROCESS:** Describe the plans for how the consensus process will be undertaken | ^*^Automatically voted in |
| **25** | **METHODS/CONSENSUS PROCESS/PARTICIPANT INFORMATION:** Describe what information will be presented to participants at the start of the consensus process | (0/8) 0% OUT |
| **26** | **METHODS/CONSENSUS PROCESS/PARTICIPANT REQUIREMENTS:** Describe what each participant will be asked to do at each stage of the consensus process | (0/8) 0% OUT |
| **27** | **METHODS/CONSENSUS PROCESS/PARTICIPANT FEEDBACK:** Describe how the participants will receive any feedback during the consensus process [it was agreed that this was not a separate item but the ‘feedback’ should be included as part of how information is summarised – see item below] | Agreed to include as part of a later item only |
| **28** | **METHODS/CONSENSUS PROCESS/NON-RESPONSE:** Describe how non-response (or partial response) will be handled during the consensus process [it was agreed to create a new item for missing data issues that includes non-response and attrition (below)] | Agreed to create a new item for missing data issues |
| **29** | **METHODS/CONSENSUS PROCESS/USER FRIENDLY MATERIAL:** Describe how the study material will be tailored for stakeholder groups such that it is understandable | (0/8) 0% OUT |
| **30** | **METHODS/CONSENSUS PROCESS/VALIDATING:** Describe any plans for validating the COS | (0/7) 0% OUT |
| **31** | **METHODS/CONSENSUS PROCESS/VOTE ORDER:** Describe how items being voted upon/discussed will be ordered for presentation to participants (e.g. a randomized order; alphabetical order; grouped according to an outcome classification framework or domains; frequency of use in previous clinical trials) | ^**^Automatically voted out |
| **32** | **METHODS/CONSENSUS PROCESS/STATEMENT OF SUPPORT:** Describe any plans for obtaining a statement of support by the stakeholders for the COS (e.g. at the end of the COS x% of participants agreed to support this COS) | ^**^Automatically voted out |
| **33** | **METHODS/CONSENSUS PROCESS/NUMBER COS:** Describe whether the investigators expect to include a particular number of outcomes in the final COS | ^**^Automatically voted out |
| **34** | **METHODS/CONSENSUS PROCESS/PARTICIPANT CHARACTERISTICS:** Describe which participant characteristics will be collected | (0/7) 0% OUT |
| **35** | **METHODS/CONSENSUS PROCESS/COS REVIEW:** Describe plans for subsequent review of the COS | ^**^Automatically voted out |
| **36** | **METHODS/CONSENSUS PROCESS/CONFLICTS-PARTICIPANTS:** Describe how potential conflicts of interest among invited participants will be identified and dealt with | (0/8) 0% OUT |
| **37** | **METHODS/CONSENSUS DEFINITION:** Describe the consensus definition | ^*^Automatically voted in |
| **38** | **METHODS/CONSENSUS DEFINITION/ADDED/COMBINED/DROPPED:** Describe the procedure for determining how outcomes will be added/combined/dropped from consideration during the consensus process | ^*^Automatically voted in |
| **39** | **ANALYSIS/SCORING-SUMMARISED:** Describe how outcomes will be scored and summarised [to include how the results are fed back to participants during the consensus process] | ^*^Automatically voted in |
| **40** | **ANALYSIS/RESPONSE RATE:** Describe how the response rate will be maximised | (1/8) 13% OUT |
| **41** | **ANALYSIS/ATTRITION:** Describe how attrition bias will be assessed [it was agreed to create a new item for missing data issues that includes attrition and none-response (above)] | Agreed to create a new item for missing data issues |
| **42** | **ANALYSIS/SORTWARE:** Describe any software that will be used during the consensus process and to analyse the results | (0/7) 0% OUT |
| **43** | **ETHICS/DISSEMINATION/ETHICS:** Describe any plans for obtaining research ethics committee / institutional review board approval in relation to the consensus process (if relevant) [agreed to combine with informed consent] | Agreed to combine with ethics approval below |
| **44** | **ETHICS/DISSEMINATION/INFORMED CONSENT:** Describe how informed consent will be obtained (if relevant) [agreed to combine with ethics approval] | Agreed to combine with informed consent above |
| **45** | **ETHICS/DISSEMINATION/CONFIDENTIALITY:** Describe any details about how the confidentiality of data collection will be preserved during the consensus process (if relevant) | (0/8) 0% OUT |
|  | ***THE ITEM BELOW WAS INTRODUCED AT THE CONSENSUS MEETING ONLY*** |  |
| **46** | **DISSEMINATION PLAN:** Describe any details about how the confidentiality of data collection will be preserved during the consensus process (if relevant) | ^***^(5/6) 83% IN |

^*^The consensus meeting panel were happy to include all these items without discussion or voting given the Delphi results demonstrated that at least 70% of participants scored between 7-9 (consensus in) for all stakeholder groups

^**^The consensus meeting panel were happy to exclude all these items without discussion or voting given the Delphi results demonstrated that <50% of participants scored between 7-9 for all stakeholder groups

^***^Item introduced at the end of the consensus meeting by the meeting chairperson
