# Supplementary material for: Core Outcome Set-STAndardised Protocol Items: the COS-STAP Statement
Source: Trials. 2019 Feb 11;20:116. doi: 10.1186/s13063-019-3230-x (PMC6371434; doi:10.1186/s13063-019-3230-x)

Additional file 5: Explanation and Elaboration Document for COS-STAP

**How to use this paper**

We developed this Explanation and Elaboration document using a similar format for other reporting guidelines, such as for COS-STAR (Core Outcome Set-STAndards for Reporting) [1]. To maximise the benefit of this document, we would encourage authors to read it in conjunction with the COS-STAP Statement.

We present each protocol item and explain the rationale for and importance of including the item in a COS development study protocol. We also provide relevant evidence from the literature, where available. We supplement each protocol item with published examples of good practice that covers key aspects that should be considered when addressing each protocol item.

Unlike the literature for previous guidelines, the pool of published COS protocols is small. COS developers should therefore be aware that the current published examples may be limited, and they may also want to consult the COMET Handbook [2] for better guidance on some of the methodological details concerning some of the items. For example, under Item 9, COS developers might want to refer to the COMET Handbook for plans on how to assess for attrition bias during the consensus process. If examples from published protocols are lacking, we supplement the examples with those from final published COS papers, if the examples there better represent the detail in the protocol item. We should also note that no systematic search was carried out to identify any of the examples, but they were identified by a variety of methods by the authors and the wider COS-STAP group.

**The COS-STAP Statement**

**TITLE and ABSTRACT**

**Item 1a: TITLE:** Identify in the title that the paper describes the protocol for the planned development of a COS

**Explanation:** Authors should identify their paper as a COS development protocol. Inclusion of the term “core outcome set” and “protocol” in the title will improve indexing which in turn will facilitate rapid retrieval from the literature.

**Example:** “Protocol for developing, disseminating and implementing a core outcome set for endometriosis” [3]

**Example:** “Core Outcomes in Ventilation Trials (COVenT): protocol for a core outcome set using a Delphi survey with a nested randomised trial and observational cohort study” [4]

**Item 1b: ABSTRACT:** Provide a structured summary

**Explanation:** An abstract provides the key information that enables readers to understand the scope and the methods of the planned COS development project.

Journals will often differ in their required abstract format and word length and while we do not favour a specific format over another, a structured abstract can often provide readers with more complete information, and can facilitate finding the key information more easily [5]. *Trials* and *BMJ Open* are two widely known journals that publish study protocols. *Trials* preferred abstract format is *Background, Methods/Design and Discussion* as its abstract headings, while *BMJ Open* uses *Introduction, Methods/Analysis and Ethics and Dissemination*.

A highly structured abstract for a protocol of a COS development study could include the following headings: *Background* (or Context), *Objectives* (or Purpose), *Data Sources, Stakeholder Eligibility Criteria* and *Consensus Methods*. In a simpler abstract structure, these above headings could be collapsed into the required format of any journal. For example, in the case of *BMJ Open*, *Background and Objectives* could form part of the *Introduction* while *Data Sources, Stakeholder Eligibility Criteria*, and *Consensus Methods* could be part of the *Methods/Analysis*.

Taking the highly structured abstract mentioned above, authors could use the *Background* heading to set the context for the readers and explain the importance and the rationale for why a COS is being developed in the area. Under the *Objectives* heading, they describe the purpose of the study and define what the scope [see item 3] of the COS will be. Under the *Data Sources* heading, they can summarise how they will obtain an original list of outcomes for inclusion in the consensus process. *Stakeholder Eligibility Criteria* describes which participants will be eligible to be involved in the consensus process with the option to describe how they will be identified. The *Consensus Methods* section would describe the methods that will be used to reach a consensus.

**Example (taken from the BMJ Open format)** [6]:

***INTRODUCTION***: Crohn's disease (CD) and ulcerative colitis (UC), the main forms of inflammatory bowel disease (IBD), are chronic, progressive and disabling disorders of the gastrointestinal tract. Although data from randomised controlled trials (RCTs) provide the foundation of evidence that validates medical therapy for IBD, considerable heterogeneity exists in the measured outcomes used in these studies. Furthermore, in recent years, there has been a paradigm shift in IBD treatment targets, moving from symptom-based scoring to improvement or normalisation of objective measures of inflammation such as endoscopic appearance, inflammatory biomarkers and histological and radiographic end points. The abundance of new treatment options and evolving end points poses opportunities and challenges for all stakeholders involved in drug development [*Background*]. Accordingly, there exists a need to harmonise measures used in clinical trials through the development of a core outcome set (COS) [*Objective*].

***METHODS AND ANALYSIS***: The development of an IBD-specific COS includes four steps. First, a systematic literature review is performed to identify outcomes previously used in IBD RCTs [*Data Sources*]. Second, semistructured qualitative interviews are conducted with key stakeholders, including patients, clinicians, researchers, pharmaceutical industry representatives, healthcare payers and regulators to identify additional outcomes of importance [*Data Sources, Stakeholder Eligibility Criteria*]. Using the outcomes generated from literature review and stakeholder interviews, an international two-round Delphi survey is conducted to prioritise outcomes for inclusion in the COS. Finally, a consensus meeting is held to ratify the COS and disseminate findings for application in future IBD trials [*Consensus Methods*].

#### ETHICS AND DISSEMINATION: Given that over 30 novel therapeutic compounds are in development for IBD treatment, the design of robust clinical trials measuring relevant and standardised outcomes is crucial. Standardising outcomes through a COS will reduce heterogeneity in trial reporting, facilitate valid comparisons of new therapies and improve clinical trial quality.

**INTRODUCTION**

**Item 2a: BACKGROUND:** Describe the background and explain the rationale for developing the COS, and identify the reasons why a COS is needed and the potential barriers to its implementation

**Explanation:** Readers need to understand the rationale behind the COS development study in the context of the health area and identify the reasons why a COS is needed. Lack of consensus between experts on what outcomes to measure can lead to inconsistencies in the reporting of outcomes, heterogeneity of outcome measurements and outcome reporting bias, all of which are valid reasons for using a consensus-based approach to develop a COS. If a COS already exists in the area, then the authors should tell the readers why they plan to develop a new COS. For example, while the new planned COS is in the same area as an existing COS, the scope (see item 3) may be different in terms of the stage of the disease for example. Alternatively, there may be known limitations or quality concerns associated with an existing COS. As an example, key minimum standards [7] may not have been considered in the development process. A good COS is one that is implemented and leads to improved outcomes for patients. Any potentially known barriers to implementation should be identified at the protocol stage with any plans on how these will potentially be overcome during the COS development process.

As an ideal background or introduction that describes the development plans of a COS, readers might consider the following. First, the authors might want to describe known problems with outcome selection and measurement in relation to the health area for which the COS is to be developed. Second, authors might want to describe the importance of a COS from different stakeholder perspectives (e.g. health care professionals, patients). Third, authors might want to add in the study aims and discuss the extent to which the limitations of any existing COS in the area, and any perceived barriers to potential implementation, will be overcome in this current study.

**Example:** Context – Development of a core outcome set for people living with dementia at home in their neighbourhoods and communities [8]

“It is currently estimated that there are 850,000 people living with dementia in the UK, two-thirds of whom live in their own homes….

In recent years, a limited consensus has been reached about what outcomes should be measured in dementia services and studies. For example, in 2007, the International Psychogeriatric Association published a consensus statement calling for clear predefined outcome measures when assessing treatment benefits for dementia. The association recommended that outcomes could include: the effect of interventions on people living with dementia’s cognition, behavioural and psychological symptoms; quality of life; global assessments and activities of daily living. In addition, it was recommended that outcomes could encompass the effects on care partners [*problems with outcome selection*].

…many completed and ongoing Cochrane reviews of interventions for people living with dementia face a high degree of variation in outcome measures. Limited consistency between studies can lead to marked heterogeneity and reporting biases thus impeding comparison of findings across studies and making meta-analyses and interpretation of results difficult [*problems with outcome selection*].

Furthermore, existing measures may not detect, or include, outcomes that are important and meaningful to people with dementia, whose perspectives are often not represented. More generally, although recent evidence suggests patient or public involvement in identifying priorities and outcomes of importance is still an emerging area, it is often not done or implemented poorly [*importance of stakeholders*]…

The central aim of work programme... is to develop a core outcome set (COS) that can be used when evaluating non-pharmacological health and social care community-based interventions for people living with dementia at home and in their own neighbourhood locality. As such, the emphasis is on identifying outcomes that are important to the person living with dementia [*study aims*].

**Item 2b: OBJECTIVES:** Describe the specific objectives with reference to developing a COS

**Explanation:** The objectives are the questions that the COS development study was designed to answer. In the context of planning a COS development study, the objectives will often refer to a consensus based approach for establishing important outcomes for a particular health condition.

**Example:** Context - development of an infant feeding core outcome set for childhood obesity interventions [9]

The aim of this study is to develop an infant feeding COS that can be used in randomized controlled studies of infant feeding interventions to prevent childhood obesity, which include infants < 1 year old. This will be done based on a systematic review of the peer-reviewed extant literature; a meeting involving multiple stakeholder perspectives to discuss and clarify outcomes; an e-Delphi survey involving an expert panel of international stakeholders; and a nominal group technique (NGT) consensus meeting to finalise the COS.

The objectives of this study are:

1. To identify all potential infant-feeding outcomes for infants up to 1 year of age in the extant literature
2. To achieve consensus on a COS for infant feeding of infants up to 1 year of age using the Delphi and nominal group techniques

**Example:** Context - development of a core outcome set for hidradenitis suppurativa (HS) trial outcomes [10]

“Study objectives are:

1. To identify a list of items and domains previously used in studies on the management of HS by a systematic review of the literature.
2. To develop a list of items relevant to HS disease severity generated by patients.
3. To develop a list of items relevant to HS disease severity generated by HS experts.
4. To combine the results of (1)—(3) into a unified list of candidate HS disease severity items for HS trials and use these to formulate potential core domains.
5. To achieve consensus on core domains for trials by in-person consensus meetings and online Delphi surveys including patient and HCP representatives at all stages.”

**Item 3: SCOPE**

**Explanation:** The scope of a COS refers to the specific area of health or health care to which the COS is to be applied [11]. The scope should be described in terms of the health condition and target population, interventions that the COS is to be applicable to and the context of use for which the COS is to be applied. The scope therefore covers the first three elements of the PICO (Population, Intervention, Comparator, Outcomes) structure for clinical research.

Defining the scope of the COS can be challenging. Ensuring it is clear, however, is recommended from the outset as this will add clarity to all stages of the COS development process, e.g. when deciding candidate outcomes to potentially include in the COS, assisting with outcome prioritisation and/or discussions in consensus meetings. If the scope is defined and agreed early in the process (i.e. at the protocol stage), it will likely reduce discussions at a late stage and possibly the need to repeat earlier work (it there was ambiguity about the scope).

**Item 3a: SCOPE (health condition and population):** Describe the *health condition(s)* and *population(s)* that will be covered by the COS

The health condition and population covered by the COS should be adequately described in the protocol. For example in cancer, the COS may cover cancer generally or it may be specific to a particular cancer type such as head and neck cancer. Similarly, a COS may be developed for all patients but it could also be developed for a specific subset of the population in mind, such as localised prostate cancer patients or advanced prostate cancer patients.

**Item 3b: SCOPE (interventions):** Describe the *intervention(s)* covered by the COS

The types of interventions covered by the COS should be adequately described in the protocol.  A COS may be developed to apply to all interventions for a particular condition or for a specific intervention, but details about what is actually covered should be provided.  As an example, if the COS is only relevant to specific classes of intervention, such as surgery or specific drug types (e.g. biologics), this detail should be presented.

**Item 3c: SCOPE (context of use):** Describe the *context of use* for which the COS is to be applied

The focus of many COS development projects is about application in clinical effectiveness trials.  However COS are applicable in other settings such as for use in other research designs, systematic reviews, routine care or audit or a COS for both clinical and research purposes.  It is important for COS developers to describe the context for which the COS is to be used in the protocol as outcomes may be different for different settings.

**Example of Scope (covering items 3a, 3b and 3c)**

**Example:** Context - development of a core outcome set for anal cancer [12]

#### Health condition

Squamous cell carcinoma of the anus/anal canal.

#### Population

Adults >18 years of age.

#### Types of interventions

Primary treatment with radiotherapy with or without concurrent chemotherapy.

#### Setting [context of use]

Later phase trials that will inform clinical decision making.

**METHODS**

**Item 4: STAKEHOLDERS:** Describe the stakeholder groups to be involved in the COS development process, the nature of and rationale for their involvement, and a description of how the individuals will be identified; this should cover both involvement as members of the research team, and as participants in the study.

**Explanation:** Knowledge of the types of stakeholder groups to be used in the COS development process is essential for appraising the COS, and therefore it is important to identify them before the consensus process begins. Describing how individuals of stakeholder groups will be identified is also important as it indicates if the list of those targeted is inclusive. During the peer review of a COS development protocol, editors and reviewers can identify any missing key stakeholder groups or make recommendations for how to reach individuals within those stakeholder groups before the COS development projects starts. Provision of these details is therefore recommended wherever possible at the protocol stage.

**Example:** Study Background: systematic review of outcomes, semi-structured interviews, two-round Delphi survey followed by a consensus meeting looking to develop a COS in inflammatory bowel disease.

“We aim to recruit a diverse participant pool, with involvement from each major stakeholder group, including patients, clinicians, researchers and representatives from patient advocacy groups, industry and research funding organisations…. During the systematic review, a list of authors with at least 25 publications in the field of IBD over the past 10 years (2006–2016), including at least two clinical trials or one systematic review of clinical trials on IBD will be compiled and invited to participate…. Clinicians experienced in managing IBD will be recruited through convenience sampling. Specifically, clinical medical and surgical leads of dedicated IBD centres from North America, Europe and the Asia-Pacific will be identified and recruited… Patients will be eligible for inclusion in the Delphi survey if they have a confirmed history of CD [*Crohn’s Disease*] or UC [*ulcerative colitis*], attendance of healthcare for IBD and fluent understanding of written English. Patients will be identified through national and international patient advocacy groups and authors’ connections. Strong collaborative partnerships between the authorship team and IBD centres in Europe and the Asia-Pacific will aim to incorporate multinational patient representation. Representatives from the pharmaceutical industry will also be invited to participate.” [6]

**Item 5a: INFORMATION SOURCES:** Describe the information sources that will be used to identify the list of outcomes. Outline the methods or reference other protocols/papers.

**Explanation:** Generating an initial list of candidate outcomes for potential inclusion is an important component of COS development. Reporting this list enables the reader to determine if outcomes considered in the consensus process are likely to be representative of the perspectives of relevant stakeholders. In a COS development protocol, authors should describe the different information sources that are to be used to generate the initial list of outcomes. For example, a list of outcomes from published clinical trials may be supplemented by undertaking interviews with patients or obtaining input from an advisory group whose membership reflects the key stakeholders. Outcomes reported from a review of trials may be mostly reflective of outcomes from a health professional’s perspective while interviews with patients might reflect outcomes that are more important to them. Developing a methodological approach to capture both healthcare professionals’ and patients’ views is particularly important in order for the COS development team to adhere to the minimum standards for COS development [7].

**Example:** Context – systematic review, focus groups, two-round Delphi and consensus meeting for the development of a core outcome set for efficacy and effectiveness trials in posterior segment-involving uveitis (PISU).

“A systematic review will be conducted on the effectiveness of pharmacological agents for PSIU and potential outcomes will be identified for inclusion in the COS... Potential outcomes to be included in the COS will be identified through focus groups with patients and carers, and one-to-one telephone interviews with health professionals, health policy-makers and commissioners”. [13]

**Item 5b: INFORMATION SOURCES:** Describe how outcomes may be dropped/combined; with reasons

**Explanation:** COS developers may drop or combine outcomes from the initial list if outcomes are considered to be overlapping in content or repetitious (Item 5a). It is necessary to provide details on how this process will be undertaken and by whom. It is important that these details are documented at the protocol stage to ensure that a process is in place to avoid losing any potentially important outcomes from the consensus process, and that any combining of outcomes is agreed for example, by the wider steering group.

**Example:** “Outcomes will be grouped under appropriate outcome domains. The outcome domains will be determined following a review of the extracted outcomes [*from a systematic review*] by the authors. The outcome domains and included outcomes will be reviewed by the Study Advisory Group to assess suitability of the domain name and grouping of outcomes.” [14]

*Note – the below examples are not from a COS protocol but from a final COS report. However, the examples provides adequate text for this protocol item.*

**Example:** “Because we expected a large diversity in reported outcomes [*from the systematic review of outcomes*], we grouped similar outcomes into overarching outcome categories by a small-group consensus process. The group of experts consisted of 6 doctors in dental surgery specialized in pediatric dentistry, including 3 clinical research investigators. First, the group identified outcomes that were identical despite different terms used across trials. Second, different but close outcomes (i.e., outcomes that could be compared across studies or combined in a meta-analysis) were grouped together into outcome domains. Finally, the group, with consensus, determined several outcome categories and produced a reduced-outcome inventory.” [15]

**Example:** Context- pain outcomes were identified for inclusion into a Delphi study through three sources: a systematic review, structured interviews and four focus groups.

“Data about the assessment of pain were extracted from these sources and coded into pain features. Coding was performed in duplicate by two researchers and pain features were then refined and modified through further discussion and review. The resulting 68 pain features were further refined through discussion with a patient and public involvement group specializing in musculoskeletal research [*Patient Experience Partnership in Research; PEP-R*] and the Project Steering Committee.” [16]

**Item 6: CONSENSUS PROCESS:** Describe the plans for how the consensus process will be undertaken

**Explanation:** There is currently variation in the approaches used to achieve consensus [17] and research to identify optimal methods for developing COS is ongoing. Methods include the Delphi technique [18, 19], nominal group technique [20, 21], consensus development conference [22] and semi-structured group discussion [23] amongst others. Many studies have used a combination of methods to reach consensus, for example, Ruperto (2003) used the Delphi approach followed by the nominal group technique [24]. Therefore it is important for authors to describe the consensus process methodology in full at the protocol stage. A flow diagram may also help describe the consensus process as in the second example below.

**Example:** “These data [*from a systematic review and interviews of patients who have experienced delirium and family members*] will inform a two-stage [*Delphi survey*] consensus process. We will hold a consensus meeting using nominal group technique to finalise outcomes for inclusion”. [25]

**Example:** (Taken from Additional File 2 of [14])


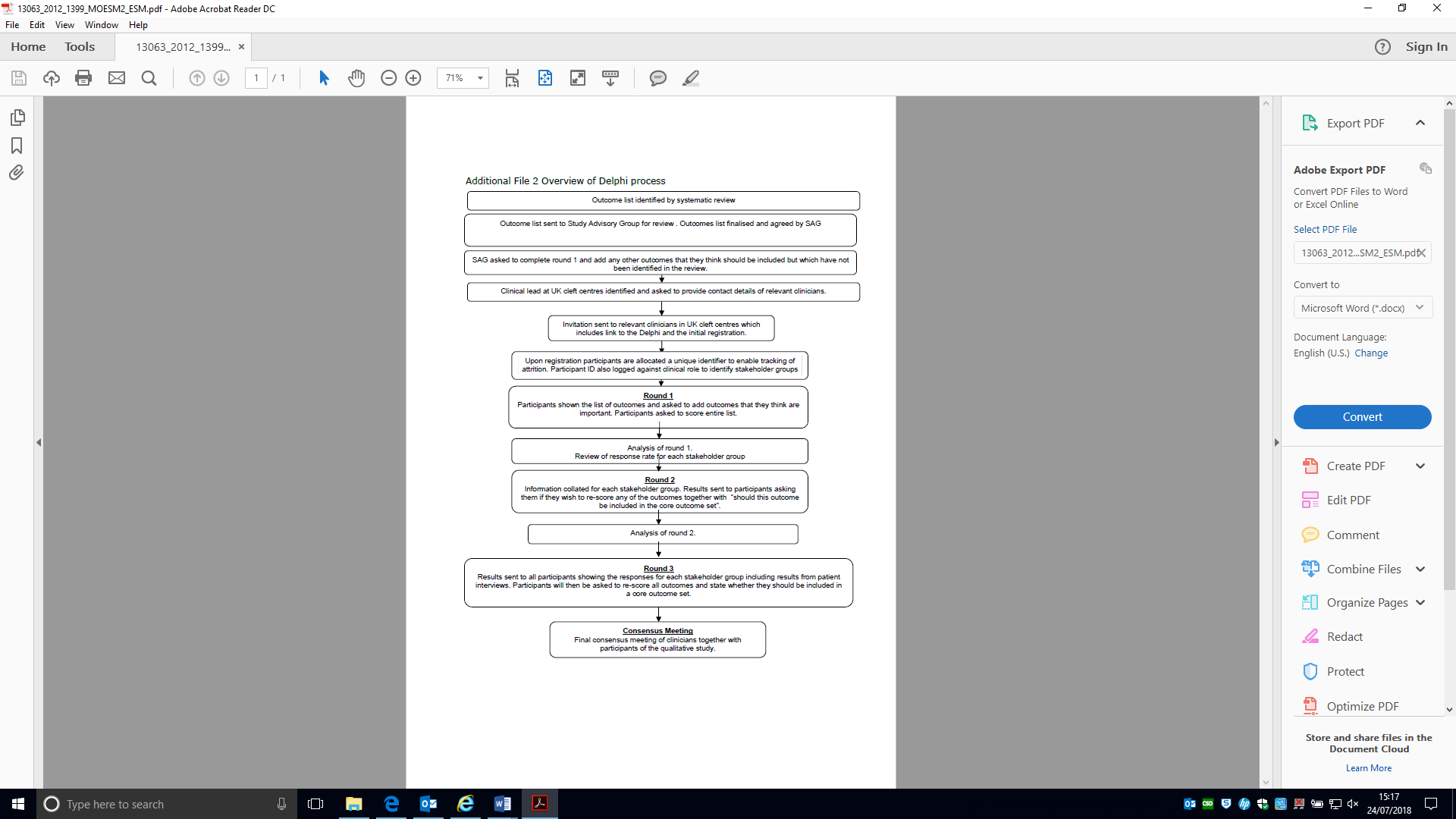


**Item 7a: CONSENSUS DEFINITION:** Describe the consensus definition

**Explanation:** The choice of what consensus criteria to use is an important consideration in COS development. Authors should define the consensus criteria a priori in the COS development protocol to minimise any potential bias from changing the criteria after the results have been analysed.

There are numerous ways proposed to define the consensus criteria, although the choice of criteria is rarely justified in the published literature [26]; commonly these relate to a threshold of percentage agreement or a mean or median threshold value for each outcome [16; 27--30]. Regardless of the consensus criteria that are to be used, authors should clearly describe the consensus definition and justify this choice where possible in the protocol. As part of an ‘issues to consider’ paper, Williamson et al 2012 describe the rationale for a ‘70/15%’ consensus definition which was used in the first example provided below [11].

**Example:** (Adapted from Table 2 of [14])

| Consensus classification | Description | Definition |
| --- | --- | --- |
| Consensus in | Consensus that outcome  should be included in  the core outcome set | 70% or more participants  scoring as 7 to 9 AND  <15% participants scoring  as 1 to 3 |
| Consensus out | Consensus that outcome  should not be included in  the core outcomes set | 70% or more participants  scoring as 1 to 3 AND <15%  of participants scoring  as 7 to 9 |
| No consensus | Uncertainty about  importance of outcome | Anything else |

**Example:** Context – COS development for all types of cardiac surgery effectiveness trials (3-Round Delphi where response options were: (a) “yes”, (b) “no” and (c) “unsure/I do not know”).

“As in round two, consensus will be set at 67 % for the panel to agree on the core domains to be included in the core domain set” [31].

**Item 7b: CONSENSUS DEFINITION:** Describe the procedure for determining how outcomes will be added/combined/dropped from consideration during the consensus process

**Explanation:** Different approaches can be used to include or exclude outcomes from consideration during the consensus process, but there is currently no empirical evidence to suggest whether the different approaches impact on the final COS. For this reason we strongly recommend that authors describe any pre-specified criteria for excluding outcomes during the consensus process, and provide the reasons for these criteria in the COS development protocol. Authors should also state criteria for which outcomes are to be carried forward throughout the whole consensus process. Authors may also plan to add in additional outcomes during the consensus process if new outcomes are suggested which were not identified during the initial outcome generation process. Authors should describe the processes for introducing new outcomes and how these new outcomes will be considered throughout the remainder of the process.

**Example:** Context – COS development for all types of cardiac surgery effectiveness trials (3-Round Delphi where response options were: (a) “yes”, (b) “no” and (c) “unsure/I do not know”).

“Our study team established a priori that domains for which at least 60 % of the participants choose the response option “no” and less than 20 % choose the response option “yes” will be dropped from the list of potential core domains. Additional outcome domains identified by participants in round one will be included if suggested by at least two participants.” [31]

**Example:** “Additional outcomes listed by participants will be reviewed and coded by two members of the study team to ensure they represent new outcomes. If there is uncertainty then the Study Management Group will be consulted, and the SAG [*scientific advisory group*] as appropriate... All outcomes will be carried forward to round 2”. [14]

**ANALYSIS**

**Item 8: OUTCOME SCORING/FEEDBACK:** Describe how outcomes will be scored and summarised, describe how participants will receive feedback during the consensus process

**Explanation:** A variety of different scoring systems have been used in COS studies to rate the importance of outcomes. The majority of studies have used Likert scales [27-30; 32] although others have used ranking of outcomes [24, 33] and allocation of points [34-35]. For transparency purposes, it is important to describe how the scoring will be done at the protocol stage to avoid any potential biases that may result from changing the scoring method during the consensus process. In the protocol, authors should tell the readers how the scoring method used signifies the level of importance of an outcome and if appropriate, describe any differences in the scoring method used for different stakeholder groups. In many consensus exercises, the results are often summarised and fed back to participants to re-consider. The results may also be summarised for use at a consensus meeting. The method of summarising and feeding back the results should also be described. This is particularly important as recent research has shown (that in a Delphi survey), the method of feedback may influence the final core set [36].

**Example:** “Participants will be asked to rank each outcome on a scale from 1 to 9… Scores of 1–3 indicate an outcome that is not important for inclusion, scores of 4–6 indicate an outcome important but not critical for inclusion and scores of 7–9 indicate an outcome felt critical for inclusion in the COS. An option to select ‘Unsure of significance’ will also be available….

Responses from round one will be analysed and collated into a feedback report. Descriptive statistics will be used to summarise the number of participants scoring each outcome and the distribution of scores…. In round two, each participant will be provided with the number of respondents and distribution of scores for each efficacy and safety outcome from the first round, stratified by stakeholder group” [6].

**Item 9: MISSING DATA:** Describe how missing data will be handled during the consensus process

The two main sources of missing data in a COS development consensus process are non-response (attrition) and partial response. Attrition bias will occur when the participants that do not respond in subsequent rounds (e.g. for a Delphi survey) have different views from their stakeholder group peers who continue to participate. For example, if the feedback a participant receives suggests that they are in a minority with regard to their scoring of importance about particular outcomes, then they may be more likely to drop out, leading to over-estimation of the degree of consensus in the final results. COS developers should also plan how they will deal with partial responders, for example, in a Delphi survey would these partial responders be invited to take part in a subsequent round or not. Developers should also specify if any criteria will be set in the protocol, e.g. partial responders will be invited in to the next round of the survey if they complete at least x% of the survey in the previous round. Authors should describe how these types of missing data will be dealt with during the consensus process in the protocol and if possible, should consider ways in which missing data can be minimised, and the potential for attrition bias can be examined.

**Example:** Context - Development of a core outcome set for medication review in older patients with multimorbidity and polypharmacy (Consensus method – Delphi survey followed by a consensus meeting).

“Participants who did not participate in Round 1 were not invited for Round 2” [37].

**Example:** Context - Development of a core outcome set for the success of gastroschisis treatment (Consensus method – three-phase Delphi survey followed by a consensus meeting).

“Bias from loss of experts between rounds will be assessed by determining if there is any difference in median round 1 scores for each outcome measure between experts who have completed both phases and experts who only completed phase 1. All outcomes will be carried forward to phase 3” [38].

**ETHICS AND DISSEMINATION**

**Item 10: ETHICS AND INFORMED CONSENT:** Describe any plans for obtaining research ethics committee / institutional review board approval in relation to the consensus process and describe how informed consent will be obtained (if relevant).

**Explanation:** Consideration of ethical and consent issues is important for any medical research and an ethics statement is often a requirement for journal submission. It is imperative that these issues are considered at the protocol stage before the COS development process begins. The level of regulatory requirements for a COS study is variable and may depend on the country where the research is undertaken or the methods and stakeholder groups that are to be involved in the consensus process.

At the COS development protocol stage, authors should describe any plans for obtaining ethical approval and detail how any informed consent issues will be handled. Authors should reference any ethical approvals if they have already been obtained, and should declare if ethical approval is not needed for their study.

**Example:** “We have received research ethics board approval from the University of Toronto (no 34296) and will seek others as required by local governance. We will obtain written consent from participants in interviews and consensus meetings. Participation in Delphi rounds will be considered indicative of consent. Consent will emphasise the voluntary nature of participation and anonymity” [25].

**Example:** “Ethical approval has been granted by the South West—Frenchay Research Ethics Committee (ref 17/SW/0025 IRAS 221625). All participants involved will be asked for their consent before participation in the qualitative interviews and the Delphi survey, and the study will be conducted according to the Declaration of Helsinki. [39]

**Example:** “The East of Scotland Research Ethics Service (EoSRES) confirmed that NHS ethical approval would not be required for this project. The University of Dundee Schools of Nursing and Health Sciences and Dentistry Research Ethics Committee (SREC) provided institutional ethical approval in December 2016 (Ref: 2016028_Lamont). Informed consent will be obtained from all participants when they opt in to participate in the study at the start of the e-Delphi process”. [40]

**Item 11: DISSEMINATION:** Describe any plans to communicate the results to study participants and COS users, inclusive of methods and timing of dissemination.

**Explanation:** A plan to disseminate COS results to study participants and COS users should be outlined in the protocol, including a process and timeframe for approving and submitting the agreed COS for dissemination (e.g. via journal publication, conference attendance, study website, relevant societies). Knowing the medium and timescales for dissemination of the COS results is particularly important for COS users who may wish use the COS in a planned new study.

**Example:** Context – development of COS for effectiveness trials of interventions to prevent and/or treat delirium

“Knowledge users within our investigator team and the support of three international Delirium Societies (American, Australasian and European) will be instrumental in dissemination of the COSs and subsequent uptake. We will provide a one page summary (clinicians/researchers and in lay language for patients and families) to these Societies for distribution among their networks and engage with them to seek additional opportunities to present our findings (educational seminars/workshops). We will disseminate our findings through peer-reviewed and open access publications and presentations at international conferences purposefully selected to reach a wide range of knowledge users taking into account geographical locations. We will engage with journal editors and funding agencies to promote awareness of our COSs”….Study timeline as follows [25]:

| Key project milestones | Start date | End date |
| --- | --- | --- |
| Systematic review | May 2017 | February 2018 |
| Patient and family member interviews | October 2017 | February 2018 |
| Delphi consensus and nested methodological study | April 2018 | March 2019 |
| Consensus meeting | June 2019 | June 2019 |
| Process evaluation patient/family interviews | February 2018 | March 2020 |
| Consensus on measures | September 2019 | March 2020 |
| Knowledge translation/dissemination | June 2019 onwards |  |

**Example:** Context – development of COS for all types of cardiac surgery effectiveness trials

“To increase COS uptake, it is recommended that developers consider engagement with the relevant Cochrane Review Groups, clinical guideline developers, research funders, journal editors, regulators such as research ethics committees, and trial registries. We intend to do so in close cooperation with the COMET Initiative. In addition, this COS and its development process will be introduced at a COMET meeting and at least at one national and international conference with a focus on cardiac surgery. The eDelphi study started in March 2015. We intend to publish the results of the eDelphi, the core domain set, in late 2015”. [31]

**ADMINISTRATIVE INFORMATION**

**Item 12: FUNDING:** Describe sources of funding, role of funders

**Explanation:** Authors of COS studies, like those of any other research study, should disclose any funding they have received to develop the COS, or state if no funding was received. Given the potential role of funding bodies as key stakeholders in the COS development process (e.g. authorities and industry representatives), it is important to be transparent about funding and the role of funders, if any, at the beginning of the COS development process. A funder may provide a service, such as the venue for a consensus meeting, with the involvement of representatives of the funding body involved in planned consensus meeting discussions. Any level of funding or service provided to the COS development team should be reported. Authors should also declare whether the funder has a role in the design and/or analysis and/or interpretation of the COS development study.

**Example:** “[Author] is funded by a National Institute for Health Research (NIHR) professorship. [Author] is funded by an NIHR doctoral research fellowship. The views expressed are those of the authors and not necessarily those of the NHS, the NIHR or the Department of Health. The NIHR had no role in the design and conduct of the study; the collection, management, analysis and interpretation of the data; the preparation, review and approval of the manuscript; or the decision to submit the manuscript for publication. [41]

**Item 13: CONFLICTS OF INTEREST:** Describe any potential conflicts of interest within the study team and how these will be managed.

**Explanation:** In addition to funding issues (Item 12), authors should report any perceived conflicts of interest related to their role (or that of other members of the study team) at the start of the COS development process which might be seen as potentially influencing the final COS. For example, a study author may have developed a measurement instrument for one of the outcomes that is likely to be considered in the consensus process. In this situation, the authors might pre-specify that such individuals be excluded from the final vote for that outcome. In some situations, the study team members may also exclude themselves from the consensus process, if it is felt that conflicts arise as a result of their involvement in the study design or involvement in generating the initial list of outcomes (Item 5a).

Authors should declare if there are no known conflicts of interest.

**Example: “**Owing to their involvement in the study design and Delphi exercise, members of the JDM Minimal Dataset Steering Committee were not asked to participate in the Delphi surveys themselves, but encouraged maximal engagement and participation of individuals within the international research groups that they represented”. [42]

**References**

[1] Kirkham JJ, Gorst S, Altman DG, Blazeby JM, Clarke M, Devane D et al. Core Outcome Set – Standards for Reporting: The COS-STAR Statement. *PLoS Medicine* 2016; **13**(10):e1002148

[2] Williamson PR, Altman DG, Bagley H, Barnes KL, Blazeby JM, Brookes ST et al. The COMET Handbook: version 1.0. *Trials* 2017; **18**(Suppl 3):280

[3] Hirsch M, Duffy JMN, Barker C, Hummelshoj L, Johnson NP, Mol B et al. [Protocol for developing, disseminating and implementing a core outcome set for endometriosis](https://bmjopen.bmj.com/content/6/12/e013998). *BMJ Open* 2016; 6 (12): e013998

[4] Blackwood B, Ringrow S, Clarke M, Marshall J, Rose L, Williamson P et al. Core Outcomes in Ventilation Trials (COVenT): protocol for a core outcome set using a Delphi survey with a nested randomised trial and observational cohort study. Trials 2015; **16**:368

[5] Liberati A, Altman DG, Tetzlaff J, Mulrow C, Gøtzsche PC, Ioannidis JPA, et al. The PRISMA Statement for Reporting Systematic Reviews and Meta-Analyses of Studies That Evaluate Health Care Interventions: Explanation and Elaboration. *PLoS Medicine* 2009; 6(7): e1000100.

[6] [Ma C](https://www.ncbi.nlm.nih.gov/pubmed/?term=Ma%20C%5BAuthor%5D&cauthor=true&cauthor_uid=28601837), [Panaccione R](https://www.ncbi.nlm.nih.gov/pubmed/?term=Panaccione%20R%5BAuthor%5D&cauthor=true&cauthor_uid=28601837), [Fedorak RN](https://www.ncbi.nlm.nih.gov/pubmed/?term=Fedorak%20RN%5BAuthor%5D&cauthor=true&cauthor_uid=28601837), [Parker CE](https://www.ncbi.nlm.nih.gov/pubmed/?term=Parker%20CE%5BAuthor%5D&cauthor=true&cauthor_uid=28601837), [Khanna R](https://www.ncbi.nlm.nih.gov/pubmed/?term=Khanna%20R%5BAuthor%5D&cauthor=true&cauthor_uid=28601837), [Levesque BG](https://www.ncbi.nlm.nih.gov/pubmed/?term=Levesque%20BG%5BAuthor%5D&cauthor=true&cauthor_uid=28601837) et al. Development of a core outcome set for clinical trials in inflammatory bowel disease: study protocol for a systematic review of the literature and identification of a core outcome set using a Delphi survey. [*BMJ Open*](https://www.ncbi.nlm.nih.gov/pubmed/28601837) 2017; 7(6): e016146.

[7] Kirkham JJ, Davis K, Altman DG, Blazeby JM, Clarke M, Tunis S et al. Core Outcome Set STAnDards for Development: The COS-STAD Recommendations. *PLoS Medicine* 2017; 14(11): e1002447.

[8] Harding AJE, Morbey H, Ahmed F, Opdebeeck C, Wang YY, Williamson PR et al. Developing a core outcome set for people living with dementia at home in their neighbourhoods and communities: study protocol for use in the evaluation of non-pharmacological community-based health and social care interventions. *Trials* 2018; 19(1):247

[9] Matvienko-Sikar K, Byrne M, Kelly C, Toomey E, Hennessy M, Devane D et al. Development of an infant feeding core outcome set for childhood obesity interventions: study protocol. Trials 2017; 18:463.

[10] Thorlacius L, Ingram JR, Garg A, Villumsen B, Esmann S, Kirby JS et al. Protocol for the development of a core domain set for hidradenitis suppurativa trial outcomes. *BMJ Open* 2017;7:e014733.

[11] Williamson PR, Altman DG, Blazeby JM, Clarke M, Devane D et al. Developing core outcome sets for clinical trials: issues to consider. *Trials* 2012; 13:132.

[12] Fish R, Sanders Ca, Williamson PR, Renehan AG. Core outcome research measures in anal cancer (CORMAC): protocol for systematic review, qualitative interviews and Delphi survey to develop a core outcome set in anal cancer. *BMJ Open* 2017; 7:e018726

[13] Tallouzi MO, Mathers JM, Moore DJ, Murray PI, Bucknall N, Blazeby JM et al. COSUMO: study protocol for the development of a core outcome set for efficacy and effectiveness trials in posterior segment-involving uveitis. Trials 2017; **18**:576.

[14] Harman NL, Bruce IA, Callery P, Tierney S, Sharif M et al. MOMENT-management of otitis media with effusion in cleft palate: protocol for a systematic review of the literature and identification of a core outcome set using a Delphi survey. *Trials* 2013; 14:70. doi:10.1186/1745-6215-14-70.

[15] Smaïl-Faugeron V, Chabouis H, Durieux P, Attal JP, Muller-Bolla M et al. Development of a core set of outcomes for randomized controlled trials with multiple outcomes – example of pulp treatments of primary teeth for extensive decay in children. *PLoS One* 2013; 8(1):e51908.

[16] Wylde V, MacKichan F, Bruce J, Gooberman-Hill R. Assessment of chronic post-surgical pain after knee replacement: Development of a core outcome set. *European Journal of Pain* 2015; 19: 611–20.

[17] Gargon E, Gurung B, Medley N, Altman DG, Blazeby JM et al. Choosing Important Health Outcomes for Comparative Effectiveness Research: A Systematic Review. *PLoS ONE* 2014; 9(6): e99111.

[18] Karas J, Ashkenazi S, Guarino A, Lo Vecchio A, Shamir R et al. A core outcome set for trials in acute diarrhoea. *Arch Dis Child*. 2015; 100: 359-63.

[19] Chiarotto A, Deyo RA, Terwee CB, Boers M, Buchbinder R et al (2015). Core outcome domains for clinical trials in non-specific low back pain. *Eur Spine J*; 24(6): 1127-42.

[20] Haywood KL, Griffin XL, Achten J, Costa ML. Developing a core outcome set for hip fracture trials. *The Bone and Joint Journal* 2014; 96-B:1016–23.

[21] Rider LG, Giannini EH, Brunner HI, Ruperto N, James-Newton L et al. International consensus on preliminary definitions of improvement in adult and juvenile myositis. *Arthritis Rheum* 2004; 50(7):2281-90.

[22] Kirchhof P, Auricchio A, Bax J, Crijns H, Camm J et al. Outcome parameters for trials in atrial fibrillation: executive summary. *European Heart Journal* 2007; 28(22):2803-2817.

[23] Zannad F, Garcia AA, Anker SD, Armstrong PW, Calvo G et al. Clinical outcome endpoints in heart failure trials. A European society of cardiology heart failure association consensus document. *Eur J Heart Fail* 2013; 15(10):1082-94.

[24] Ruperto N, Ravelli A, Murray KJ, Lovell DJ, Andersson-Gare B et al. Preliminary core sets of measures for disease activity and damage assessment in juvenile systemic lupus erythematosus and juvenile dermatomyositis. *Rheumatology* 2003; 42(12):1452-9.

[25] Rose L, Agar M, Burry LD, Campbell N, Clarke M, Lee J et al. Development of core outcome sets for effectiveness trials of interventions to prevent and/or treat delirium (Del-COrS): study protocol. *BMJ Open* 2017;7:e016371.

[26] Diamond IR, Grant RC, Feldman BM, Pencharz PB, Ling SC, Moore AM, Wales PW. Defining consensus: a systematic review recommends methodologic criteria for reporting of Delphi studies. Journal of Clinical Epidemiology 2014; 67(4):401-9.

[27] Blazeby JM, Macefield R, Blencowe NS, Jacobs M, McNair AG et al. Core information set for oesophageal cancer surgery. British Journal of Surgery 2015; 102(8): 936-43.

[28] Potter S, Holcombe C, Ward JA, Blazeby JM. Development of a core outcome set for reconstructive breast surgery The BRAVO (Breast Reconstruction and Valid Outcomes) Study. *British Journal of Surgery* 2015; 102 (11): 1360-71.

[29] Bennett WL, Robinson KA, Saldanha IJ, Wilson LM, Nicholson WK. High priority research needs for gestational diabetes mellitus. *Journal of Women’s Health* 2012; 21(9):925-932.

[30] Schmitt J, Langan S, Stamm T, Williams HC. Core outcome set domains for controlled trials and clinical recordkeeping in eczema: international multiperspective Delphi consensus process. *Journal of Investigative Dermatology* 2011; 131(3):623-630.

[31] [Moza A](https://www.ncbi.nlm.nih.gov/pubmed/?term=Moza%20A%5BAuthor%5D&cauthor=true&cauthor_uid=26625730), [Benstoem C](https://www.ncbi.nlm.nih.gov/pubmed/?term=Benstoem%20C%5BAuthor%5D&cauthor=true&cauthor_uid=26625730), [Autschbach R](https://www.ncbi.nlm.nih.gov/pubmed/?term=Autschbach%20R%5BAuthor%5D&cauthor=true&cauthor_uid=26625730), [Stoppe C](https://www.ncbi.nlm.nih.gov/pubmed/?term=Stoppe%20C%5BAuthor%5D&cauthor=true&cauthor_uid=26625730), [Goetzenich A](https://www.ncbi.nlm.nih.gov/pubmed/?term=Goetzenich%20A%5BAuthor%5D&cauthor=true&cauthor_uid=26625730). A core outcome set for all types of cardiac surgery effectiveness trials: a study protocol for an international eDelphi survey to achieve consensus on what to measure and the subsequent selection of measurement instruments. [*Trials*](https://www.ncbi.nlm.nih.gov/pubmed/26625730) 2015;16:545.

[32] Harman NL, Bruce IA, Kirkham JJ, Tierney S, Callery P et al. The importance of integration of stakeholder views in core outcome set development: otitis media with effusion in children with cleft palate. *PLoS ONE* 2015; **10**(6): e0129514.

[33] Vargus-Adams JN, Martin LK. Measuring What Matters in Cerebral Palsy: A Breadth of Important Domains and Outcome Measures. *Archives of Physical Medicine and Rehabilitation* 2009*;* 90:2089-2095.

[34] Kloppenburg M, Bøyesen P, Smeets W, Haugen IK, Liu R et al. Report from the OMERACT hand osteoarthritis special interest group: advances and future research priorities. *J Rheumatol* 2014; 41(4): 810-8.

[35] Mease PJ, Clauw DJ, Arnold LM, Goldenberg DL, Witter J et al. Fibromyalgia syndrome. See comment in PubMed Commons below[*J Rheumatol.*](http://www.ncbi.nlm.nih.gov/pubmed/16265715) 2005; 32(11):2270-7.

# [36] Brookes ST, Macefield RC, Williamson PR, McNair AG, Potter S, Blencowe N et al. Three nested randomized controlled trials of peer-only or multiple stakeholder group feedback within Delphi surveys during core outcome and information set development. *Trials* 2016; 17:409.

[37] Beuscart J-B, Dalleur O, Boland B, Thevelin S, Knol W, Cullinan S et al. Development of a core outcome set for medication review in older patients with multimorbidity and polypharmacy: a study protocol. *Clinical Interventions in Aging* 2017; 12: 1379-1389

# [38] Allin B, Ross A, Marven S, Hall NJ, Knight M. Development of a core outcome set for use in determining the overall success of gastroschisis treatment. *Trials* 2016; 17: 360.

# [39] Young A, Brookes S, Rumsey N, Blazeby J. Agreement on what to measure in randomised controlled trials in burn care: study protocol for the development of a core outcome set. *BMJ Open* 2017; 7:e017267

# [40] Lamont TJ, Clarkson JE, Ricketts DNJ, Heasman PA, Ramsey CR. Core outcomes in periodontal trials: study protocol for core outcome set development. *Trials* 2017; 18:436

# [41] Allin B, Bradnock T, Kenny S, Walker G, Knight M. NETS^1HD^: study protocol for development of a core outcome set for use in determining the overall success of Hirschsprung’s disease treatment. *Trials* 2016; 17:577

[42] McCann LJ, Kirkham JJ, Wedderburn LR, Pilkinson C, Huber AM, Ravelli A, Appelbe D, Williamson PR, Beresford MW. Development of an internationally agreed minimal dataset for juvenile dermatomyositis (JDM) for clinical and research use. Trials 2015; **16**:268

#
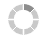

Supplement: Supplementary file 5 — Explanation and Elaboration document for COS-STAP. (DOCX 253 kb) [file 13063_2019_3230_MOESM5_ESM.docx]
